# Melatonin inhibits bladder tumorigenesis by suppressing PPARγ/ENO1-mediated glycolysis

**DOI:** 10.1038/s41419-023-05770-8

**Published:** 2023-04-06

**Authors:** Dexin Shen, Zhao Deng, Wei Liu, Fenfang Zhou, Yayun Fang, Danni Shan, Gang Wang, Kaiyu Qian, Mengxue Yu, Yi Zhang, Lingao Ju, Yu Xiao, Xinghuan Wang

**Affiliations:** 1 Department of Urology, Zhongnan Hospital of Wuhan University, Wuhan, China; 2 Department of Urology, Aerospace Center Hospital, Peking University Aerospace School of Clinical Medicine, Beijing, China; 3 Department of Radiology, Zhongnan Hospital of Wuhan University, Wuhan, China; 4 Department of Biological Repositories, Zhongnan Hospital of Wuhan University, Wuhan, China; 5 Human Genetic Resources Preservation Center of Hubei Province, Wuhan, China; 6 Euler Technology, ZGC Life Sciences Park, Beijing, China; 7 Center for Quantitative Biology, School of Life Sciences, Peking University, Beijing, China; 8 Wuhan Research Center for Infectious Diseases and Cancer, Chinese Academy of Medical Sciences, Wuhan, China

**Keywords:** Bladder cancer, Diagnostic markers, Targeted therapies

## Abstract

Melatonin is a well-known natural hormone, which shows a potential anticancer effect in many human cancers. Bladder cancer (BLCA) is one of the most malignant human cancers in the world. Chemoresistance is an increasingly prominent phenomenon that presents an obstacle to the clinical treatment of BLCA. There is an urgent need to investigate novel drugs to improve the current clinical status. In our study, we comprehensively explored the inhibitory effect of melatonin on BLCA and found that it could suppress glycolysis process. Moreover, we discovered that ENO1, a glycolytic enzyme involved in the ninth step of glycolysis, was the downstream effector of melatonin and could be a predictive biomarker of BLCA. We also proved that enhanced glycolysis simulated by adding exogenous pyruvate could induce gemcitabine resistance, and melatonin treatment or silencing of *ENO1* could intensify the cytotoxic effect of gemcitabine on BLCA cells. Excessive accumulation of reactive oxygen species (ROS) mediated the inhibitory effect of melatonin on BLCA cells. Additionally, we uncovered that PPARγ was a novel upstream regulator of ENO1, which mediated the downregulation of ENO1 caused by melatonin. Our study offers a fresh perspective on the anticancer effect of melatonin and encourages further studies on clinical chemoresistance.

## Introduction

Bladder cancer (BLCA) is a malignant tumor originating from the bladder mucosa and is one of the top ten common tumors in humans [1]. According to the latest data, in 2022, the number of estimated new BLCA cases in China is 91,893, and in the United States, it is 84,825, which makes BLCA the second most common cancer of the human urinary system [2]. BLCA can occur at any age, even in children. Its incidence rate increases with age, with a higher incidence at the ages of 50 to 70 years. The incidence rate of BLCA in men is three to four times more than in women [1, 2]. Moreover, compared with the data published earlier, the number of estimated new BLCA cases and deaths is on the rise [1–5]. The high incidence and mortality rate render BLCA a global “killer”, threatening public health. Treatment of BLCA has transformed from traditional binary therapy consisting of surgery and chemotherapy, to modern multidimensional therapy consisting of surgery, chemotherapy, radiotherapy, and immunotherapy [6–8]. Nevertheless, clinical drug resistance is an unavoidable dilemma in patients treated with cisplatin and gemcitabine. Therefore, we need to investigate the mechanism underlying clinical drug resistance and explore novel drugs to elevate or treat chemosensitivity in the current BLCA treatment strategy.

Melatonin (N-acetyl-methoxy-tryptamine) is a well-known anti-inflammatory and antioxidant natural hormone synthesized in the human pineal gland [9]. However, melatonin administered at a pharmacological concentration range inhibits multiple human cancers and can synergistically interact with the currently used drugs [10–14]. In breast cancer, melatonin promotes the degradation of HER2 and increases the efficiency of lapatinib [15, 16]. In acute myeloid leukemia, melatonin sensitizes FLT3/ITD-positive cells to sorafenib by re-modifying the cellular redox homeostasis [17]. In renal cell carcinoma, melatonin initiates “tumor slimming” by promoting lipid browning and autophagy mediated by the PGC1α/UCP1 axis [18]. In prostate cancer, melatonin reduces lipid accumulation by restoring the expression level of CES1, a tumor suppressor, and reversing enzalutamide resistance in castration-resistant prostate cancer (CRPC) animal models [19]. In head and neck squamous cell carcinoma, melatonin modulates folate metabolism by suppressing the MTHFD1L-formate axis [20]. Moreover, melatonin amplifies the cytotoxic effect of shikonin by promoting the excessive accumulation of reactive oxidative species (ROS) and disrupting the SIRT3-SOD2-AKT pathway in several human cancer cells [10]. Nevertheless, no research is available on the anticancer effect of melatonin on human BLCA, especially whether melatonin could modulate the metabolic program in BLCA and therefore suppress the progression of BLCA, and the subject deserves further exploration.

In this study, we thoroughly investigated anticancer effects of melatonin on BLCA. We demonstrated that melatonin could repress glycolysis in BLCA by targeting the PPARγ-ENO1-glycolysis axis and causing ROS accumulation to disrupt cellular homeostasis. Moreover, we revealed that when melatonin interrupts glycolysis, this could elevate the sensitivity of BLCA cells to gemcitabine. Generally, our study revealed a novel metabolic reprogramming role of melatonin in treating BLCA and presented a promising prospect of melatonin in future BLCA treatment.

## Materials and methods

### Cell culture experiments

BLCA cell lines (T24, 5637, UM-UC3) and human embryonic kidney cell 293 T were purchased from the Chinese Academy of Sciences (Shanghai, China). T24 and 5637 were maintained in RPMI-1640 medium (Gibco, USA) with 10% FBS (Excell Bio, China). UM-UC3 was maintained in MEM medium (Gibco, USA) with 10% FBS. 293 T was maintained in DMEM medium (Hyclone, USA) with 10% FBS. All human cell lines have been authenticated using STR profiling. All cell lines were cultured in a humidified atmosphere with 5% CO_2_ and 95% air in a 37 °C incubator.

### Drug treatment

Melatonin (MCE, HY-B0075), GW9662 (MCE, HY-16578), and Rosiglitazone (MCE, HY-17386) were supplemented into the medium to treat BLCA cells with an annotated duration. Gemcitabine (Selleck, S1714) was supplemented into the medium for 24 h before melatonin treatment or with siRNA transfection for 48 h. Pyruvate (MCE, HY-Y0781) and N-acetyl-L-cysteine (NAC) (MCE, HY-B0215) were supplemented 8 h before melatonin treatment, gemcitabine, or siRNA transfection.

### Detection of Caspase 3 activity

Cells treated with melatonin for 24 h were collected and washed with PBS three times. All the subsequent steps were performed according to the user’s instructions for the Caspase 3 Activity Assay Kit (Beyotime, C1116).

### Detection of cellular pyruvate

1.5 × 10^5^ cells were placed per well in 6-well plates and cultured for 24 h. Then, the cells were treated with melatonin, with an annotated concentration, for 24 h or *ENO1*-specific siRNAs for 48 h. Cells were collected, and all subsequent steps were performed according to the user’s instructions for the Pyruvate Assay Kit (Abcam, ab65342).

### Dual-luciferase reporter assay

293 T cells were added to 24-well plates 24 h before transfection. pGL3-ENO1-WT and pGL3-ENO1-Mut luciferase reporter plasmids were purchased from GeneCopoeia (Guangzhou, China). JASPAR online database (https://jaspar.genereg.net/) was used for putative binding site prediction, and the binding sites were deleted to construct the pGL3-ENO1-Mut luciferase reporter plasmid. Luciferase activity was measured by a dual-luciferase reporter assay system (Promega, E1910).

### Nucleic acid transfection

*ENO1*-specific and *PPARγ*-specific siRNAs were purchased from GenePharma (Suzhou, China). PPARγ-Flag plasmid was purchased from the Miaoling plasmid platform (Wuhan, China). The sense sequence of *ENO1*, *HIF-1α* and *PPARγ* target siRNAs are listed in Supplementary Table S1.

### Total mRNA extraction and qRT-PCR

Total mRNA extraction was performed according to the user’s instructions for the HiPure Total RNA Mini Kit (Magen, R4111-03). qRT-PCR was performed using iTaq Universal SYBR Green Supermix (Bio-Rad, #1725125). The primer sequences are listed in Supplementary Table S2.

### Chromatin immunoprecipitation (ChIP) assay

1 × 10^7^ cells were cross-linked using 1% methanal and were ultrasonicated for chromatin fragmentation. PPARγ or IgG antibodies were cocultured with fragmented chromatin for 12 h at 4°C. Proteinase K was added for de-crosslinking, and samples were incubated for 12 h in a water bath at 65°C. Then, qRT-PCR was performed for quantification. All the subsequent steps were performed according to the user’s instructions for the SimpleChIP Plus Sonication Chromatin IP Kit (CST, #56383). The primers and antibodies used in this study are listed in Supplementary Tables S2 and S3, respectively.

### Gene set enrichment analysis (GSEA)

GSEA version 4.2.3 was used to analyze RNA-seq data of melatonin treatment and CCLE transcription data. Normalized enrichment score (NES) > 1 and *p* < 0.05 were considered significant.

### RNA-seq analysis

Total RNA was extracted as mentioned above, and further treated with DNase to remove genomic DNA contamination. Isolation of mRNA was performed using the NEBNext PolyA mRNA Magnetic Isolation Module (NEB, USA), and the mRNA was then used for RNA-Seq library preparation with the NEBNext Ultra Directional RNA Library Prep Kit for Illumina (NEB, USA). The library was then subjected to Illumina sequencing with paired-end 2*150 bp as the sequencing mode.

Raw reads were filtered to obtain high-quality clean reads by removing sequencing adapters, short reads (length < 35 bp) and low-quality reads using Cutadapt v1.18 and Trimmomatic v0.35. Then FastQC is used to ensure high reads quality. The clean reads were mapped to the human genome (assembly GRCh38) using the HISAT2 v2.1.0 software.

### Western blot

Cells were lysed in RIPA (Beyotime, P0013B) buffer containing protease and phosphatase inhibitors. Equal amounts of cell lysates were separated using SDS-PAGE. Antibodies are listed in Supplementary Table S3.

### Proliferation assays

For methyl thiazolyl tetrazolium (MTT) assay, 2500 cells per well were placed into a 96-well plate with a 200 μL medium and incubated at 37 °C. 20 μL of MTT solution was added per well, and the cells were incubated for 4 h. Then, the medium was discarded, and 200 μL DMSO was added to dissolve the precipitate. The microplate reader (SpectraMax M2, USA) detected an absorption value of 570 nm. For the clone formation assay, 1000 cells per well were placed into a six-well plate with 2 mL medium. T24 and 5637 cells were cultured for 10 days, and UM-UC3 cells were cultured for 7 days. 4% PFA was used to fix colonies for 1 h, and 0.1% crystal violet was used to stain colonies for 1 h.

### Flow cytometry of cell cycle, apoptosis, and ROS

Cells were collected and washed three times with PBS. For the cell cycle analysis, cells were re-suspended in 1 mL DNA staining solution. 10 μL propidium iodide (PI) solution (MultiSciences Biotech, China) was added. The cells were incubated in darkness for at least 30 min. For the apoptosis analysis, cells were re-suspended in 100 μL 1× Annexin V binding buffer, and then, we sequentially added 5 μL Annexin V-FITC and 5 μL PI solution (Sungene Biotech, China). Another 400 μL 1× Annexin V binding buffer was added after 30 min of incubation. A parameters regulation was performed at each independent experiment according to the user’s instructions. For the ROS analysis, cells were re-suspended in 1 mL FBS-free medium, and 1 μL DCFH-DA (Sigma, D6883) was added. After 30 min of incubation, cells were centrifuged and re-suspended in a 1 mL FBS-free medium. Each sample was analyzed using flow cytometry analysis (Beckman, USA).

### Migration assays

For the transwell assays, 40,000 UM-UC3 or T24 or 120,000 5637 BLCA cells per well were placed into the upper chamber with an FBS-free medium. The bottom chambers were supplemented with a medium containing 10% FBS. After being incubated for 24 h in the incubator, cells that stayed in the upper membrane were discarded, and cells that crossed the membrane were fixed and stained, and pictures were taken.

### In vivo experiments

The Experimental Animal Welfare and Ethics of the Zhongnan Hospital of Wuhan University (approval No. ZN2021253) approved this study. Male BALB/c nude mice (4 weeks of age) were purchased from WTLH Co., Ltd. (Beijing, China). T24 BLCA cells were used to construct the in vivo model infected by LV-*shENO1* and LV-*shNC*. The sequence for LV-*shENO1* and LV-*shNC* were 5’-GCAUUGGAGCAGAGGUUUATT-3’ and 5’-UUCUCCGAACGUGUCACGUTT-3’, respectively. All mice were adaptively fed for 7 days in SPF environment and then were randomly divided. The investigator was blinded to the group allocation of the animals during the experiment. The sample size of each experiment is shown in the legend. No data were excluded from the analysis.

For the subcutaneous tumor-bearing experiment, 4 × 10^6^ infected T24 cells in 100 µL PBS were subcutaneously injected into the bilateral abdomen of each mouse. Drugs were intraperitoneally injected. The tumor size was measured every 5 days on the 10th day after injection. The tumor size was calculated as follows: (length × width^2^)/2 (mm^3^). Melatonin was given at a dose of 100 mg/kg, and gemcitabine was given at a dose of 10 mg/kg every 2 days. Forty-five days after the injection, mice were sacrificed, and the xenograft tumors were removed and weighed.

For the lung metastasis experiment, 9 × 10^5^ infected T24 cells in 100 µL PBS were injected into the tail vein. Melatonin was given at a dose of 100 mg/kg. Forty-five days after the injection, the mice were photographed and sacrificed, and the lungs were removed for photographing and staining.

### Immunohistochemistry (IHC) and Hematoxylin & Eosin (H&E) staining

For H&E staining, we put the slices in xylene I for 10 min, xylene II for 10 min, absolute ethanol I for 5 min, absolute ethanol II for 5 min, 95% alcohol for 5 min, 90% alcohol for 5 min, 80% alcohol for 5 min, and 70% alcohol for 5 min and washed them with distilled water. The slices were treated with the following and washed with distilled water after each treatment: harris hematoxylin stain for 3–8 min, 1% hydrochloric acid in 70% alcohol for a few seconds for differentiation, and 0.6% ammonia. We stained the slices with eosin staining solution for 1–3 min. We put the slices in 95% alcohol I for 5 min, 95% alcohol II for 5 min, absolute ethanol I for 5 min, absolute ethanol II for 5 min, xylene I for 5 min, and xylene II for 5 min, dehydrate and transparent, take the slices out of xylene and dry them slightly, and seal them with neutral gum. Microscopic examination, image acquisition, and analysis.

IHC staining was performed as previously reported [21].

### Statistical analysis

All experiments were performed at least three times. Two-tailed Student’s *t* test was used to evaluate the statistical significance of differences between the two groups. We used GraphPad Prism Version 8.0 to perform the statistical analysis. *p < 0.05, **p < 0.01, and ***p < 0.001 were considered statistically significant.

### Reporting summary

Further information on research design is available in the Nature Research Reporting Summary linked to this article.

## Results

### Melatonin inhibited BLCA proliferation and metastasis

**Fig. 1:**
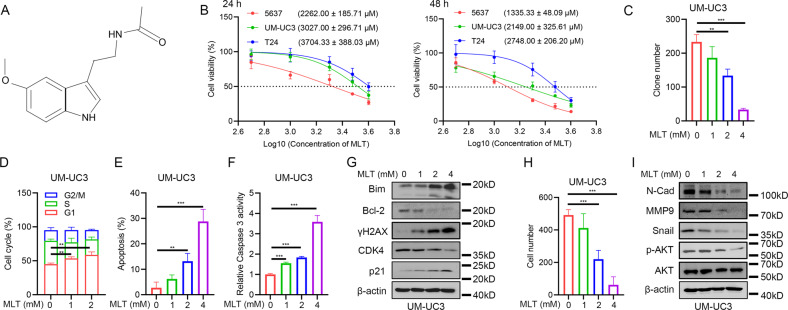
Melatonin exerted inhibitory effects on proliferation and metastasis of BLCA cells. **A** Structural of melatonin. **B** IC50 value of 24 h and 48 h melatonin treatment on BLCA cells (*n* = 3). **C** Statistical analysis of clone formation assay of UM-UC3 cells under 48 h melatonin treatment (*n* = 3). **D** Statistical analysis of cell cycle distribution of UM-UC3 cells under 24 h melatonin treatment (*n* = 3). **E** Statistical analysis of apoptotic cells of UM-UC3 cells under 24 h melatonin treatment (*n* = 3). **F** Caspase 3 activity assay of UM-UC3 cells under 24 h melatonin treatment (*n* = 3). **G** Western blot assay of apoptosis-related and cell cycle-related proteins of UM-UC3 cells after 24 h melatonin treatment. **H** Transwell assay of UM-UC3 cells after 24 h melatonin treatment and statistical analysis. **I** Western blot assay of EMT-related proteins cells after 24 h melatonin treatment. *p < 0.05, **p < 0.01, ***p < 0.001.

We first performed proliferation assays to examine the sensitivity of BLCA cells to melatonin (Fig. 1A). The results demonstrated that the inhibitory effect of melatonin on BLCA cells is time- and dose-dependent (Fig. 1B). The clone formation assay showed that clone numbers decreased with an increase in melatonin concentration (Fig. 1C and Supplementary Fig. S1A, B). The cell cycle analysis showed that melatonin treatment caused a significant G1 phase arrest (Fig. 1D and Supplementary Fig. S2A) and a decrease in cycle-related proteins like CDK4 and an increase in p21 (Fig. 1G and Supplementary Fig. S2E). The results of apoptosis also demonstrated that the cytotoxic effect of melatonin is dose-dependent (Fig. 1E and Supplementary Fig. S2B). Besides, the detection of caspase 3 activity showed that melatonin treatment activated caspase 3 (Fig. 1F and Supplementary Fig. S2C). Western blot assays showed treatment with melatonin significantly elevated Bim, a pro-apoptosis protein, while it decreased Bcl-2, an anti-apoptosis protein. Moreover, γH2AX, a marker of DNA double-strand break, presented a profound augmentation (Fig. 1G and Supplementary Fig. S2E).

Next, we explored whether melatonin could suppress the metastatic ability of BLCA cells. The transwell assays showed that melatonin treatment decreased the migratory ability of BLCA cells in a dose-dependent manner (Fig. 1H and Supplementary Fig. S2D). The level of EMT-related proteins, including N-Cad, MMP9, and Snail, was decreased in a dose-dependent manner (Fig. 1I and Supplementary Fig. S2F). Moreover, we found that melatonin treatment inhibited the phosphorylation of AKT (Fig. 1I and Supplementary Fig. S2F). To conclude, we showed that melatonin treatment exerted a comprehensive and dose-dependent inhibitory effect on the proliferation and metastasis ability of BLCA cells by causing cell cycle arrest, inducing apoptosis, and suppressing AKT activity.

### Identification of ENO1 as a potential downstream of melatonin in human BLCA cells

**Fig. 2:**
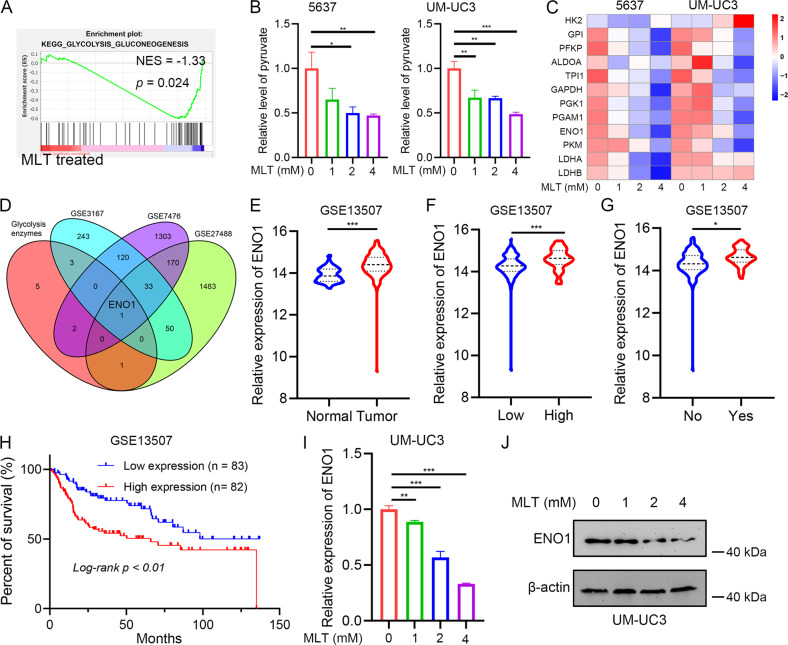
Identification of ENO1 as a potential target of melatonin in BLCA cells. **A** GSEA result of RNA-seq of BLCA cells after 24 h melatonin (0 mM, 1 mM, 2 mM, and 4 mM) treatment. **B** Detection of cellular pyruvate level after 24 h melatonin treatment in BLCA cells (*n* = 3). **C** Heatmap of the alteration of glycolytic enzymes in BLCA cells after 24 h melatonin (0 mM, 1 mM, 2 mM, and 4 mM) treatment. **D** Venn map of co-DEGs in GSE3167, GSE7476, GSE27488, and glycolysis enzymes. **E** Expression level of *ENO1* in normal bladder tissues versus BLCA tissues in GSE13507 dataset. **F** Expression level of *ENO1* in low-grade BLCA tissues versus high-grade BLCA tissues in GSE13507 dataset. **G** Expression level of *ENO1* in BLCA tissues with or without progression in GSE13507 dataset. **H** Survival analysis of BLCA patients with different *ENO1* mRNA level in GSE13507 dataset. **I** qRT-PCR analysis of *ENO1* expression alteration after 24 h melatonin (0 mM, 1 mM, 2 mM, and 4 mM) treatment in BLCA cells (*n* = 4). **J** Western blot assay of ENO1 protein alteration after 24 h melatonin (0 mM, 1 mM, 2 mM, and 4 mM) treatment in BLCA cells. *p < 0.05, **p < 0.01, ***p < 0.001.

We performed RNA-seq analysis to explore the inhibitory effect of melatonin on BLCA cells. Notably, GSEA results demonstrated that the downregulated genes were closely correlated with the glycolysis pathway (Fig. 2A), reminding us of melatonin’s potential metabolic reprogramming effect on BLCA cells. Our subsequent analysis also validated that melatonin treatment could decrease the intracellular pyruvate level in a dose-dependent manner (Fig. 2B). Heatmap results directly reflected the overall expression status of the glycolysis enzymes in BLCA cells under melatonin treatment (Fig. 2C). Moreover, we found that ENO1 was the only enzyme with a differential expression status in BLCA samples (Fig. 2D).

Then, we explored the expression status of *ENO1* in BLCA samples and found that *ENO1* was significantly upregulated in BLCA tissues compared with normal bladder tissues (Fig. 2E). High-grade BLCA tissues presented a higher *ENO1* expression than low-grade (Fig. 2F). The mRNA level of *ENO1* was positively related to BLCA progression (Fig. 2G). Chi-square test of the characteristics of BLCA samples in the GSE13507 dataset revealed that the expression level of *ENO1* is highly correlated with BLCA development (Table 1). The survival analysis demonstrated that patients with a higher *ENO1* level tend to have a shorter life span (Fig. 2H). The bioinformatics analysis proved ENO1 was a potential biomarker that could predict the progression and prognosis of BLCA. Moreover, our qRT-PCR and western blot results both demonstrated that melatonin treatment inhibited *ENO1* expression, and the effect was dependent on the concentration of melatonin (Fig. 2I–J). To conclude, we discovered that melatonin treatment could suppress the glycolysis process of BLCA cells, and the glycolytic enzyme ENO1 may mediate the inhibitory effect of melatonin on BLCA cells.

**Table 1:** Clinicopathological statistics of BLCA patients from GSE13507 based on *ENO1* expression level.

Clinicopathological Features	*ENO1* expression level		Total	*P* value
	Low	High		
Gender				
Female	10 (12.34%)	20 (23.81%)	50	0.0563
Male	71 (87.66%)	64 (76.19%)	115	
Grade				
Low	65 (80.25%)	40 (47.62%)	105	<0.001^***^
High	16 (19.75%)	44 (52.38%)	60	
Invasiveness				
Muscle invasive	21 (25.93%)	41 (48.81%)	62	0.0024^**^
Non-muscle invasive	60 (74.07%)	43 (51.19%)	103	
M stage				
M0	79 (97.53%)	79 (94.05%)	158	0.2671
M1 + MX	2 (2.47%)	5 (5.95%)	7	
N stage				
N0	77 (95.06%)	72 (85.71%)	149	0.0425*
N1 + N2 + N3 + NX	4 (4.94%)	12 (14.29%)	16	
Progression				
Yes	7 (8.64%)	24 (28.57%)	31	0.0011^**^
No	74 (91.36%)	60 (71.43%)	134	

*p < 0.05, **p < 0.01, ***p < 0.001.

### Silencing of *ENO1* inhibited the proliferation and metastasis of BLCA cells

Next, we continued to investigate the role of ENO1 in BLCA cells. We evaluated the efficiency of *ENO1*-specific siRNAs (Supplementary Fig. S3). *ENO1* deficiency significantly limited the proliferation of BLCA cells (Supplementary Fig. S4A, B). Cell cycle analysis showed that silencing of *ENO1* caused a prominent G1 phase arrest (Supplementary Fig. S4C), which was analogous to melatonin treatment. When *ENO1* was silenced, CDK4, a cell cycle-related protein, was downregulated, and p21 was upregulated (Supplementary Fig. S4E). Similarly, silencing of *ENO1* significantly induced apoptosis in BLCA cells (Supplementary Fig. S4D). Western blot assays showed that silencing of *ENO1* led to the corresponding alteration of apoptosis-related proteins, such as the elevation of Bim, the augmentation of γH2AX, and the reduction of Bcl-2 (Supplementary Fig. S4E).

Moreover, similar to the effect of melatonin treatment on BLCA cells, silencing of *ENO1* also significantly suppressed the metastasis of UM-UC3 and T24 cells (Supplementary Fig. S4F). Western blot showed that proteins participating in the EMT process decreased after *ENO1* was silenced (Supplementary Fig. S4G). Our results demonstrated that similar to the effect of melatonin treatment, *ENO1* deficiency markedly inhibited the proliferation and metastatic ability of BLCA cells.

### *ENO1* mediated the inhibitory effect of melatonin on BLCA cells

The above results have confirmed that silencing of *ENO1* could get similar results to treating BLCA cells with melatonin, so we hope to further explore the role of ENO1 in the inhibitory effect of melatonin on BLCA cells. Our results showed that silencing of *ENO1* and overexpressing of ENO1 exhibited a converse effect on melatonin treatment. On the one hand, simultaneously silencing *ENO1* and treating BLCA cells with melatonin exerted a synergetic effect. Proliferation assays showed that the growth of BLCA cells nearly stagnated (Supplementary Fig. S5A, D). The average apoptotic cells also increased dramatically (Supplementary Fig. S5F). And the migration ability of BLCA cells was also further decreased (Supplementary Fig. S5H). However, overexpressing ENO1 not only promotes the proliferation (Supplementary Fig. S5B, C and E) and migration ability of BLCA cells (Supplementary Fig. S5I), but also mitigates the inhibitory effect of melatonin. Cell viability assays showed that the sensitivity of BLCA cells was decreased when ENO1 was overexpressed (Supplementary Fig. S5C), and the reduced clone number via melatonin treatment was also rescued when ENO1 was overexpressed (Supplementary Fig. S5E). The number of apoptotic cells caused by melatonin treatment was also reversed via overexpressing ENO1 (Supplementary Fig. S5G). Moreover, the damaged migration ability of BLCA cells was also recovered when ENO1 was overexpressed (Supplementary Fig. S5I). The above results demonstrated that ENO1 mediated the inhibitory effect of melatonin on BLCA cells.

### Pyruvate reversed the inhibitory effect of melatonin and *ENO1* deficiency on BLCA cells

**Fig. 3:**
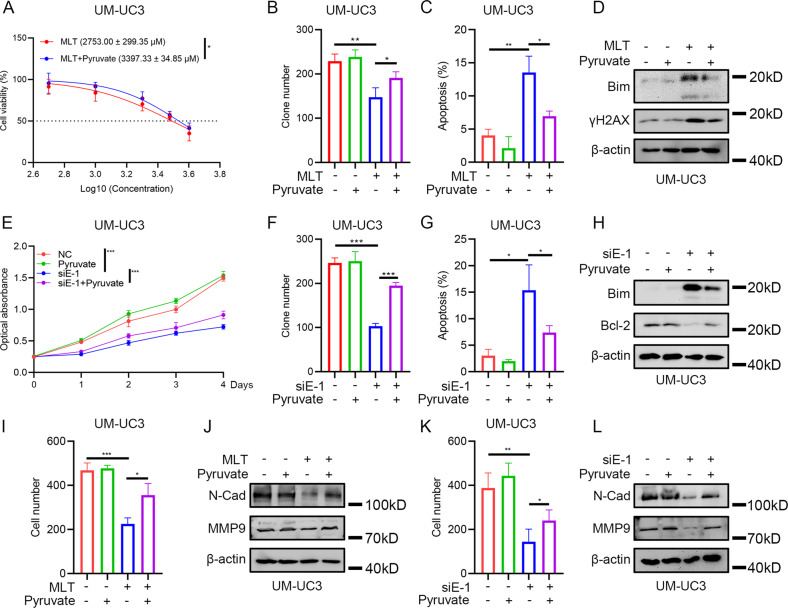
Supplement of exogenous pyruvate could reverse the inhibitory effect of melatonin treatment or silencing of *ENO1* on BLCA cells. **A** IC50 value of 24 h melatonin treatment on UM-UC3 cells with exogenous pyruvate (3 mM) supplement (*n* = 3). **B** Statistical analysis of clone formation assay of UM-UC3 cells under 48 h melatonin (2 mM) treatment and exogenous pyruvate (3 mM) supplement (*n* = 3). **C** Statistical analysis of apoptotic cells of UM-UC3 cells after 24 h melatonin (2 mM) treatment with exogenous pyruvate (3 mM) supplement (*n* = 3). **D** Western blot assay of apoptosis-related proteins of UM-UC3 cells after 24 h melatonin (2 mM) treatment with exogenous pyruvate (3 mM) supplement. **E** MTT assay of UM-UC3 cells after silencing *ENO1* with exogenous pyruvate (3 mM) supplement (*n* = 3), negative control siRNA was added. **F** Statistical analysis of clone formation assay of UM-UC3 cells after silencing *ENO1* with exogenous pyruvate (3 mM) supplement (*n* = 3), negative control siRNA was added in “-” of siE-1 group. **G** Statistical analysis of apoptotic cells of UM-UC3 cells after silencing *ENO1* with exogenous pyruvate (3 mM) supplement (*n* = 3), negative control siRNA was added in “-” of siE-1 group. **H** Western blot assay of apoptosis-related proteins of UM-UC3 cells after silencing *ENO1* with exogenous pyruvate (3 mM) supplement, negative control siRNA was added in “-” of siE-1 group. **I** Statistical analysis of transwell assay of UM-UC3 cells after 24 h melatonin (2 mM) treatment with exogenous pyruvate (3 mM) supplement and statistical analysis (*n* = 3). **J** Western blot assay of EMT-related proteins of UM-UC3 cells after 24 h melatonin (2 mM) treatment with exogenous pyruvate (3 mM) supplement. **K** Statistical analysis of transwell assay of UM-UC3 cells after silencing *ENO1* with exogenous pyruvate (3 mM) supplement (*n* = 3), negative control siRNA was added in “-” of siE-1 group. **L** Western blot assay of EMT-related proteins of UM-UC3 cells after silencing *ENO1* with exogenous pyruvate (3 mM) supplement, negative control siRNA was added in “-” of siE-1 group. *p < 0.05, **p < 0.01, ***p < 0.001.

Previous results implied a potential relationship between melatonin and the glycolysis pathway. Silencing *ENO1* also decreased the intracellular pyruvate level, which is consistent with its role as a glycolysis enzyme (Supplementary Fig. S6). Next, we tried to examine whether the enhanced glycolysis process, simulated by supplementing exogenous pyruvate, could rescue the inhibitory effect of melatonin treatment or silencing of *ENO1* on BLCA cells. The proliferation assays showed pyruvate supplementation rescued BLCA growth under melatonin treatment or silencing of *ENO1* (Fig. 3A, B, E, F and Supplementary Fig. S7A, B). Moreover, adding exogenous pyruvate rescued the number of apoptotic cells compared with using melatonin treatment or silencing of *ENO1* only (Fig. 3C, G and Supplementary Fig. S7C, D). Western blot assays demonstrated that pyruvate supplementation reduced the augmentation of Bim and γH2AX and recovered the level of anti-apoptosis protein Bcl-2 (Fig. 3D, H). The transwell assays showed that exogenous pyruvate supplementation rescued the inhibitory effect of melatonin treatment or silencing of *ENO1* on the metastatic ability of BLCA cells (Fig. 3I, K and Supplementary Fig. S7E, F). The expression levels of EMT-protein N-Cad and MMP9 were also correspondingly reversed (Fig. 3J, L). In conclusion, our rescue assays indicated that enhanced glycolysis, simulated by pyruvate supplementation, could effectively reverse the inhibitory effect of melatonin treatment or silencing of *ENO1*.

### Melatonin treatment or silencing of *ENO1* promoted the cytotoxic effect of gemcitabine

**Fig. 4:**
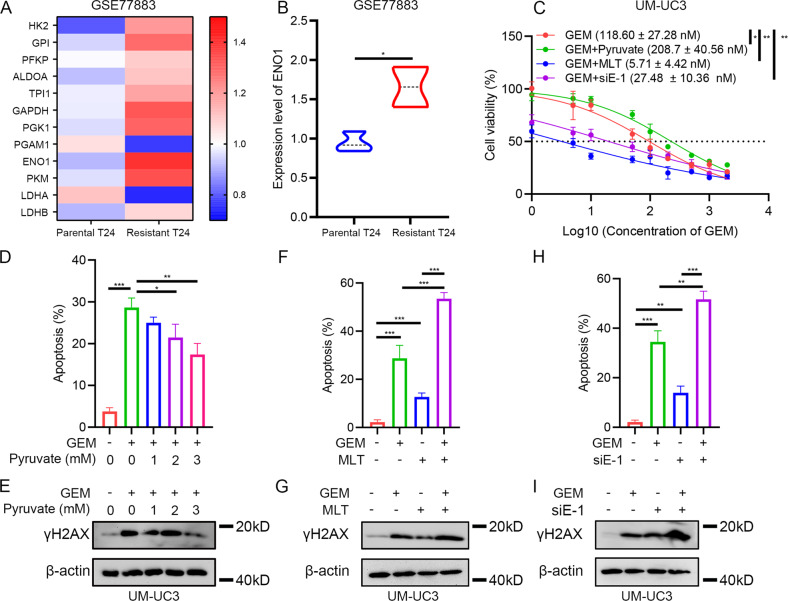
Melatonin treatment or silencing of *ENO1* could promote the cytotoxic effect of gemcitabine. **A**–**B** Expression status of glycolysis enzymes in GSE77883. **C** IC50 value of gemcitabine on BLCA cells with different culture conditions under 48 h culture (*n* = 3), negative control siRNA was added. **D** Statistical analysis of apoptotic cells of UM-UC3 cells after 48 h gemcitabine (0.5 μM) treatment with exogenous pyruvate (3 mM) supplement (*n* = 3). **E** Western blot assay of γH2AX alteration in UM-UC3 cells with 48 h gemcitabine (0.5 μM) treatment and 3 mM pyruvate supplement. **F** Statistical analysis of apoptotic cells of UM-UC3 cells with 48 h gemcitabine (0.5 μM) treatment and 24 h 2 mM melatonin treatment (*n* = 4). **G** Western blot assay of γH2AX alteration in UM-UC3 cells with 48 h gemcitabine (0.5 μM) treatment and 24 h 2 mM melatonin treatment. **H** Statistical analysis of apoptotic cells of UM-UC3 cells with 48 h gemcitabine (0.5 μM) treatment and silencing *ENO1* (*n* = 3), negative control siRNA was added in “-” of siE-1 group. **I** Western blot assay of γH2AX alteration in UM-UC3 cells with 48 h gemcitabine (0.5 μM) treatment and silencing *ENO1*, negative control siRNA was added in “-” of siE-1 group. *p < 0.05, **p < 0.01, ***p < 0.001.

Previous research have shown that enhanced glycolysis drives chemoresistance [22–25], and suppressing glycolysis could promote the efficiency of chemotherapy [26, 27]. Therefore, we investigated whether melatonin treatment could enhance traditional chemotherapy. First, we found that most glycolytic enzymes were elevated in gemcitabine-resistant T24 cells, and *ENO1* was significantly upregulated therein (Fig. 4A, B). The proliferation assays showed that exogenous pyruvate supplementation decreased the sensitivity of BLCA cells to gemcitabine (Fig. 4C and Supplementary Fig. S8A). Pyruvate supplementation reduced the percentage of apoptotic cells under gemcitabine treatment, and the cytoprotective effect of pyruvate against gemcitabine was dose-dependent (Fig. 4D and Supplementary Fig. S8B). The results of western blot analysis showed that exogenous pyruvate supplementation rescued augmented γH2AX in gemcitabine-treated BLCA cells (Fig. 4E). In contrast, proliferation assays showed that adding melatonin dramatically elevated the sensitivity of BLCA cells to gemcitabine (Fig. 4C), and the percentage of apoptotic cells was also dramatically elevated (Fig. 4F and Supplementary Fig. S8C). The level of γH2AX was further increased compared with using melatonin or gemcitabine treatment only (Fig. 4G). Similar results were observed when using *ENO1*-specific siRNA combined with gemcitabine in BLCA cells (Fig. 4C, H, I and Supplementary Fig. S8D). To conclude, we demonstrated that enhanced glycolysis limited the efficiency of gemcitabine, while suppressing the glycolysis process with melatonin treatment or silencing of *ENO1* promoted the cytotoxic effect of gemcitabine on BLCA cells.

### ROS mediated the inhibitory effect of melatonin or silencing of *ENO1* on BLCA cells

A study showed that a high concentration of melatonin induced abnormal ROS accumulation in other cancer cells [28], which is similar to our results in BLCA cells (Supplementary Fig. S9A). Also, our results showed that the induction of ROS in BLCA cells via melatonin was time-dependent (Supplementary Fig. S10A) and apoptosis-related proteins like Bim and γ-H2AX were also correspondingly changed (Supplementary Fig. S10B). Additionally, our GSEA results showed that *ENO1* was positively correlated with the ROS pathway and may induce intrinsic apoptosis by regulating oxidative stress (Supplementary Fig. S9B). Flow cytometry analysis showed that silencing of *ENO1* induced a considerable accumulation of ROS (Supplementary Fig. S9C). Next, we added NAC to the medium to perform rescue assays. Apoptosis assays revealed that adding NAC efficiently reversed cell apoptosis under melatonin treatment and silencing of *ENO1* (Supplementary Fig. S9D, F and Supplementary Fig. S11A, B). Clone formation assays showed that cell proliferation was improved with the decrease of cellular ROS accumulation (Supplementary Fig. S9E, G). Flow cytometry analysis showed that adding exogenous pyruvate alleviated the abnormal accumulation of ROS (Supplementary Fig. S9H, I). The transwell assays showed that adding NAC rescued the inhibitory effect of melatonin or silencing of *ENO1* on the metastasis of BLCA cells (Supplementary Fig. S9J, K). To conclude, we demonstrated that ROS was the main factor that mediated the suppressive effect of melatonin on BLCA cells.

### Melatonin treatment or silencing of *ENO1* suppressed BLCA growth and metastasis in vivo

**Fig. 5:**
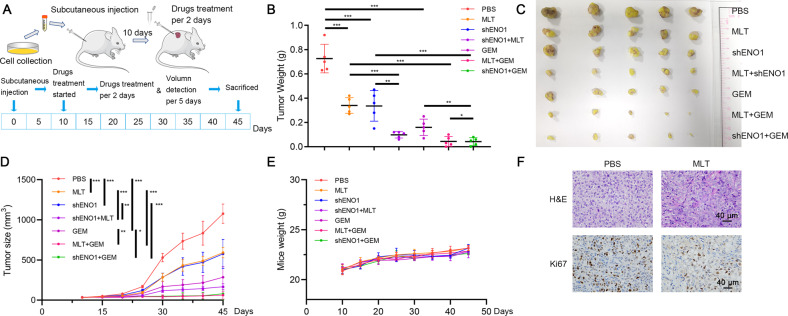
Melatonin treatment or silencing of *ENO1* could suppress BLCA growth in vivo. **A** Pattern of in vivo model construction and drug treatment. **B** Weight of xenograft tumors (*n* = 5). **C** General view of dissected tumors. **D** Volume of xenograft tumors (*n* = 5). **E** Measurement of mice weight. **F** Representative pictures of staining assays. Scale bar: 40 μm. *p < 0.05, **p < 0.01, ***p < 0.001.

We next established animal models to investigate the effect of melatonin and silencing *ENO1* on the tumorigenesis of BLCA cells in vivo (Fig. 5A). We examined the efficiency of LV-*shENO1* (Supplementary Fig. S12A, B). The results indicated that melatonin treatment or silencing of *ENO1* significantly inhibited tumor growth compared with the PBS-treated group, and combined use further reduced the tumor size (Fig. 5B–D). Moreover, melatonin treatment or silencing of *ENO1* dramatically promoted the cytotoxic effect of gemcitabine in vivo (Fig. 5B–D). There was no apparent difference in the mice’s weight among the group (Fig. 5E). The staining results showed that the Ki67 level was higher in the PBS-treated group (Fig. 5F). The lung metastasis model showed that the fluorescence intensity was significantly reduced in the melatonin-treated group compared with the PBS-treated group (Supplementary Fig. S13A, B). Dissected samples showed more visible metastatic nodules in the PBS-treated group, and H&E stain assays showed relatively minor metastatic nodules and healthier lung tissues in the melatonin-treated group (Supplementary Fig. S13C, D).

### PPARγ mediated the downregulation of ENO1 caused by melatonin

**Fig. 6:**
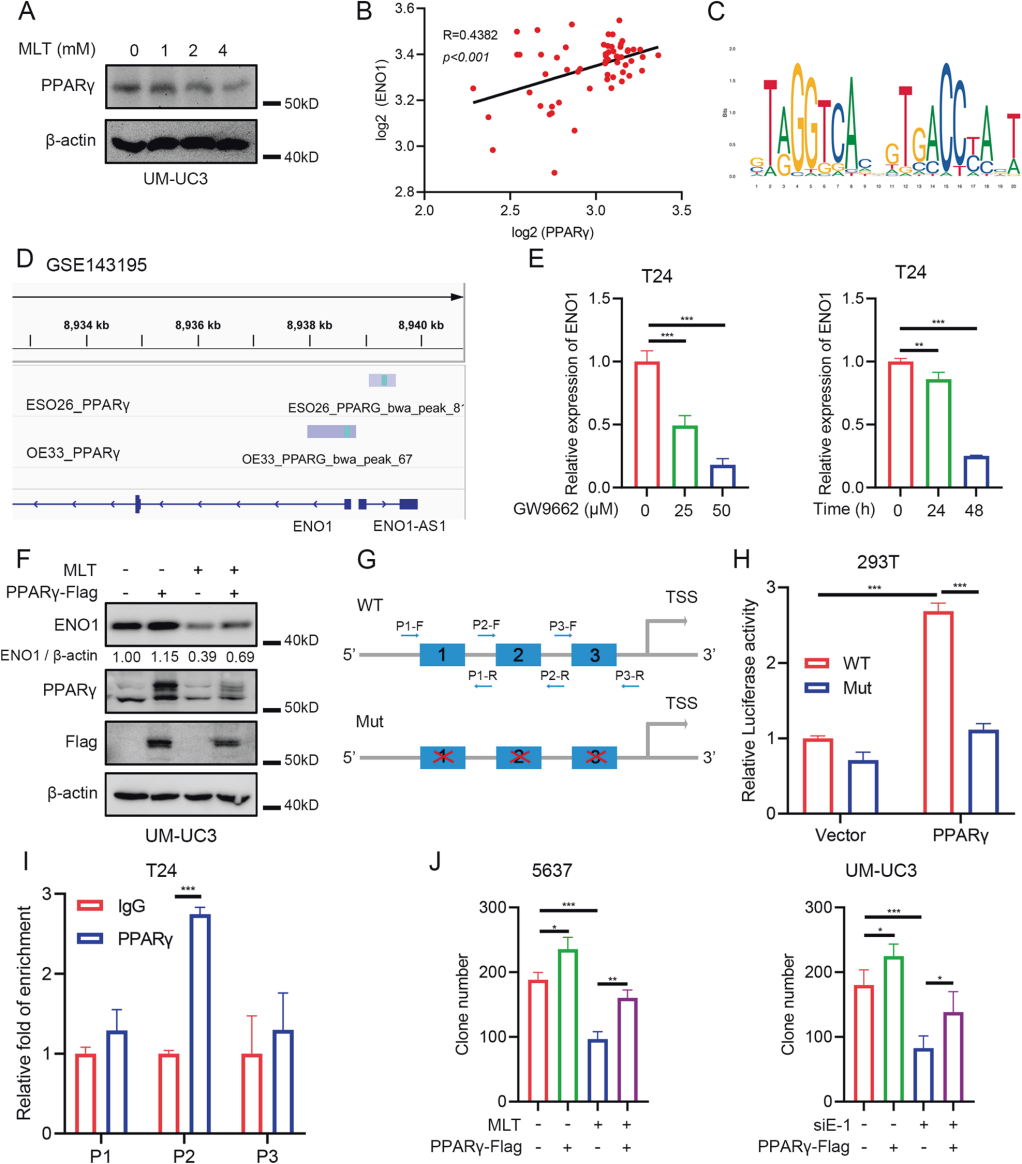
PPARγ mediated the downregulation of ENO1 by melatonin. **A** Western blot results of PPARγ protein alteration with 24 h melatonin treatment. **B** Spearman correlation analysis of the expression level of *ENO1* and *PPARγ* in GSE3167. **C** The binding site of PPARγ obtained from the JASPAR database. **D** ChIP-seq data of esophageal adenocarcinoma showing the binding of PPARγ on ENO1 promoter region. **E** qRT-PCR results of *ENO1* mRNA level with GW9662 treatment in T24 cells (*n* = 3). **F** Western blot results of ENO1 protein level with PPARγ overexpression and melatonin (4 mM) treatment, empty vector was added. **G** Pattern of dual-luciferase reporter construction and ChIP-qRT-PCR primer design. **H** Dual-luciferase reporter assay of *ENO1* promoter activity (*n* = 3). **I** ChIP-qPCR assay of putative PPARγ binding sites on *ENO1* promoter (*n* = 3). **J** Statistical analysis of clone formation assay of 5637 cells and UM-UC3 cells (*n* = 3), empty vector or negative control siRNA was added. *p < 0.05, **p < 0.01, ***p < 0.001.

We further investigated the STRING database to explore the potential regulation of ENO1. Interestingly, the results showed that ENO1 could be involved in the “HIF1α and PPARγ regulation of glycolysis” pathway (Supplementary Fig. S14A and Supplementary Table S4). To the best of our knowledge, HIF1α stayed inactive under normoxia incubation. Moreover, as PPARγ is a pivotal transcription factor in metabolism programming [29–32], we speculated that instead of HIF1α, PPARγ could be the upstream regulator that mediated the *ENO1* transcription by melatonin. To verify our hypothesis, we transfected *HIF1α* specific siRNAs to observe whether *ENO1* could be decreased under normoxia conditions. Results showed that silencing of *HIF1α* did not change the mRNA level nor the protein level of ENO1 (Supplementary Fig. S14B, C). Therefore, we excluded the possibility of HIF1α in mediating the downregulation of *ENO1* by melatonin. Western blot showed that melatonin treatment reduced the protein level of PPARγ (Fig. 6A). Pearson’s correlation analysis showed that the expression level of *PPARγ* and *ENO1* was positively correlated in BLCA datasets (Fig. 6B). Online database showed PPARγ could directly bind to the promoter region of ENO1 promoter and ChIP-seq data in esophageal adenocarcinoma showed prominent binding peaks of PPARγ on *ENO1* promoter region (Fig. 6C, D). The inhibition of PPARγ by GW9662, a potent antagonist of PPARγ, significantly decreased the mRNA level of *ENO1* (Fig. 6E). Analysis of the published data showed that the inhibition of PPARγ decreased the mRNA level of *ENO1* in BLCA cells (Supplementary Fig. S14D) [33, 34], while Rosiglitazone (a PPARγ agonist) could increase the transcription level of *ENO1* in melanoma cells (Supplementary Fig. S14E). Silencing of *PPARγ* by transfecting specific siRNAs caused the reduction of the protein level of ENO1 in BLCA cells (Supplementary Fig. S14F). Moreover, transfecting UM-UC3 cells with PPARγ-Flag plasmid reversed the downregulated ENO1 protein level under melatonin treatment (Fig. 6F). Treating BLCA cells with Rosiglitazone also effectively recovered the reduced mRNA level of *ENO1* by melatonin treatment (Supplementary Fig. S14G). Dual-luciferase reporter assays showed that PPARγ promoted the activity of wild-type ENO1 promoter plasmid (Fig. 6G, H). Both melatonin and GW9662 treatments inhibited the activity of ENO1 luciferase reporter (Supplementary Fig. S14H). ChIP-qPCR analysis showed that PPARγ presented a strong binding activity on the *ENO1* promoter (Fig. 6I). Proliferation assays showed that overexpression of PPARγ reversed the inhibitory effect of melatonin treatment or silencing of *ENO1* on the growth of BLCA cells (Fig. 6J and Supplementary Fig. S14I, J). To conclude, we dissected PPARγ as the upstream regulator that mediated the downregulation of *ENO1* expression caused by melatonin, and the overexpression of PPARγ reversed the suppressive effect of melatonin or silencing of *ENO1* on the growth of BLCA cells.

## Discussion

In tumor cells, enhanced glycolysis is one of the most prominent metabolic characteristics, which provides rapid ATP production and creates an acidic tumor microenvironment with increased lactate secretion [35, 36]. This process converts pyruvate into lactate without entering the tricarboxylic acid cycle to complete oxidative phosphorylation, reducing ROS production and protecting the mitochondria [35]. In contrast, suppressing or reversing glycolysis and switching to oxidative phosphorylation increase the burden on the mitochondria and cause mitochondrial dysfunction and excessive ROS production [37–39]. In our study, we found that ENO1, a glycolytic enzyme, was a novel downstream effector that mediated the inhibitory effect of melatonin on BLCA cells. Melatonin treatment suppresses glycolysis with a significant reduction of cellular pyruvate and causes an abnormal accumulation of ROS. Using NAC, a ROS scavenger, efficiently reduced the percentage of apoptotic cells, indicating that accumulated ROS plays a pivotal role in melatonin’s cytotoxic effect on BLCA cells. Additionally, exogenous pyruvate supplementation alleviated the accumulation of ROS and significantly rescued the proliferation and metastatic ability of BLCA cells under melatonin treatment, which verified that melatonin interrupted the glycolysis in BLCA, which led to the abnormally accumulated and fatal ROS.

Clinical chemoresistance is a prominent obstacle to the treatment quality and prognosis of cancer patients. Among the multiple theories for the mechanism that promotes the progression of chemoresistance, glycolysis occupied a pivotal position [37, 40–43]. For example, in fluoropyrimidine 5-fluorouracil (5-FU) resistant colorectal and gastric cancers, inhibiting PKM2-mediated and LDHA-mediated glycolysis significantly enhanced the cytotoxicity of 5-FU [44, 45]. Notably, Dong et al. reported that the protein level of ENO1 was upregulated in 5-FU-resistant colorectal cancer [23]. We simulated enhanced glycolysis by adding exogenous pyruvate and found that pyruvate supplementation significantly elevated the tolerance of BLCA cells and decreased the percentage of apoptotic cells under gemcitabine treatment. In contrast, both melatonin treatment and silencing of *ENO1* efficiently intensified the cytotoxicity of gemcitabine. As for how glycolysis could affect the efficiency of gemcitabine, we speculated that pyruvate may be an essential effector. Gemcitabine is phosphorylated into diphosphate and nucleoside diphosphate after entering the cells, inhibiting the DNA chain’s extension and leading to DNA breakage and cell apoptosis [46]. Previous studies reported that enhanced glycolysis promoted DNA damage repair [47–49]. Recently, Wu et al. [50] reported that pyruvate could directly bind to SSRP1, which increased the association of the facilitates chromatin transcription (FACT) complex with γH2AX and subsequently facilitated the FACT-mediated chromatin loading of γH2AX, ultimately promoting DNA repair and tumor cell survival. Serine racemase (SRR) is an enzyme that catalyzes the dehydration of l-serine and d-serine to pyruvate [51, 52]. Ohshima et al. [53] reported that SRR silencing intensified, and adding pyruvate decreased the cytotoxic effect of 5-FU on colorectal cancer cells. They indicated that this happened because pyruvate may help maintain histone acetylation since pyruvate is the main source of acetyl-CoA. Therefore, we consider pyruvate a protective metabolite that maintains cellular homeostasis. A decreased intracellular pyruvate pool caused by melatonin treatment or silencing of *ENO1* caused chromosome instability or interrupted DNA repair, intensifying the cytotoxic effect of gemcitabine.

PPARγ is a member of the nuclear receptor peroxisome proliferation activated receptor subfamily, which combines with retinoid X receptor (RXR) to form a heterodimer and regulates the transcription of metabolic genes by binding to the peroxisome proliferation response element (PPRE) [54–56]. Our group and other researchers reported the role of PPARγ in promoting BLCA progression [33, 57, 58]. PPARγ was also reported to participate in glycolysis. In hepatocellular carcinoma (HCC), PPARγ, phosphorylated at Ser84, promoted PFKFB4 transcription, thereby promoting glycolysis to maintain HCC progression [32]. Feng et al. [30] reported that simvastatin resensitized HCC to sorafenib by suppressing HIF1α/PPARγ/PKM2-mediated glycolysis. In our research, we found that melatonin treatment inhibited the protein level of PPARγ in a dose-dependent manner and PPARγ was an upstream regulator of ENO1. Rescue assays confirmed that overexpression of PPARG significantly rescued the growth of BLCA cells under melatonin treatment or silencing of *ENO1*. As for how melatonin treatment reduced the protein level of PPARγ, we speculated that the SIRT1-PPARγ negative feedback loop could function. First, SIRT1 is a well-known PPARγ suppressor that deacetylates PPARγ at Lys268 and Lys293 [59, 60], and the mRNA level of *PPARγ* was not significantly changed under melatonin treatment (Supplementary Fig. S15A), which indicated that the post-transcriptional modulation of PPARγ protein mediated the downregulation of PPARγ by melatonin. Second, Xiao et al. [18] and Zhou et al. [19] reported that melatonin treatment elevated the protein level of SIRT1 in clear cell renal cell carcinoma (ccRCC) (ccRCC) and prostate cancer (PCa). Third, we found that the mRNA level of *SIRT1* was upregulated (Supplementary Fig. S15B). Fourth, our GSEA results showed that the CAMKK2 pathway was potentially activated (data not shown), and the activated Ca^2+^-CAMKK2-AMPK axis was reported to promote *SIRT1* transcription [61, 62]. Based on the four points, we assumed that melatonin treatment induced the influx of Ca^2+^ and activated the CAMKK2-AMPK axis to promote the transcription of *SIRT1* while inducing SIRT1 repressed PPARγ protein level leading to the downregulation of *ENO1* and finally causing the interruption of glycolysis. This assumption deserves future validation.

**Fig. 7:**
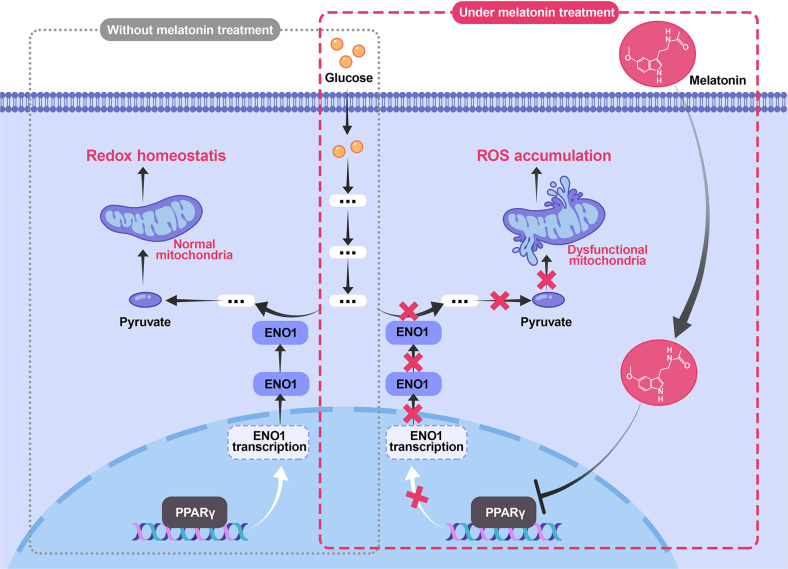
Melatonin inhibited tumorigenesis of bladder cancer via suppressing PPARγ/ENO1-mediated glycolysis. Left panel: without melatonin treatment, PPARγ promotes *ENO1* transcription to complete the integral glycolysis process, and the mitochondrion function normally to maintain cellular redox homeostasis. Right panel: under melatonin treatment, melatonin inhibits PPARγ-ENO1-mediated glycolysis. Decreased pyruvate production causes increased mitochondrion burden leading to abnormal ROS accumulation.

To conclude, we uncovered that melatonin suppressed glycolysis to inhibit the tumorigenesis of BLCA and identified that the PPARγ-ENO1 axis was the downstream effector of melatonin (Fig. 7). Moreover, we proved that, for melatonin to exert an inhibitory effect on BLCA cells, ROS was pivotal. We also confirmed that enhancing glycolysis was favorable to gemcitabine resistance, and suppressing this biological process elevated the sensitivity of BLCA cells to gemcitabine treatment. Our research offers a fresh perspective on the anticancer effect of melatonin and encourages future studies on clinical chemoresistance.

### Statement of animal rights

This study was approved by the Animal Experimental Center of Zhongnan Hospital of Wuhan University (approval No. ZN2021253).

## Supplementary information

Supplementary Figures S1-S15

Supplementary Tables S1-S4

Related file. Original Western blots

Reporting Summary

## Data Availability

BLCA sample transcription data of GSE3167, GSE7476, GSE27448, GSE13507 datasets, gemcitabine-resistant cell transcription data of GSE77883, and esophageal adenocarcinoma ChIP-seq data of GSE143195 were downloaded from GEO database (https://www.ncbi.nlm.nih.gov/gds). BLCA cell lines transcription data were downloaded from CCLE database (https://sites.broadinstitute.org/ccle). The original RNA-seq data were submitted to the GEO database at the following link, https://www.ncbi.nlm.nih.gov/geo/query/acc.cgi?acc=GSE212599.
