# Supplementary material for: Melatonin inhibits bladder tumorigenesis by suppressing PPARγ/ENO1-mediated glycolysis
Source: Cell Death Dis. 2023 Apr 6;14(4):246. doi: 10.1038/s41419-023-05770-8 (PMC10079981; doi:10.1038/s41419-023-05770-8)
Supplement: Supplementary file 1 — Supplementary Figures S1-S15 [file 41419_2023_5770_MOESM1_ESM.docx]

**Supplementary Figures S1-S15**

**
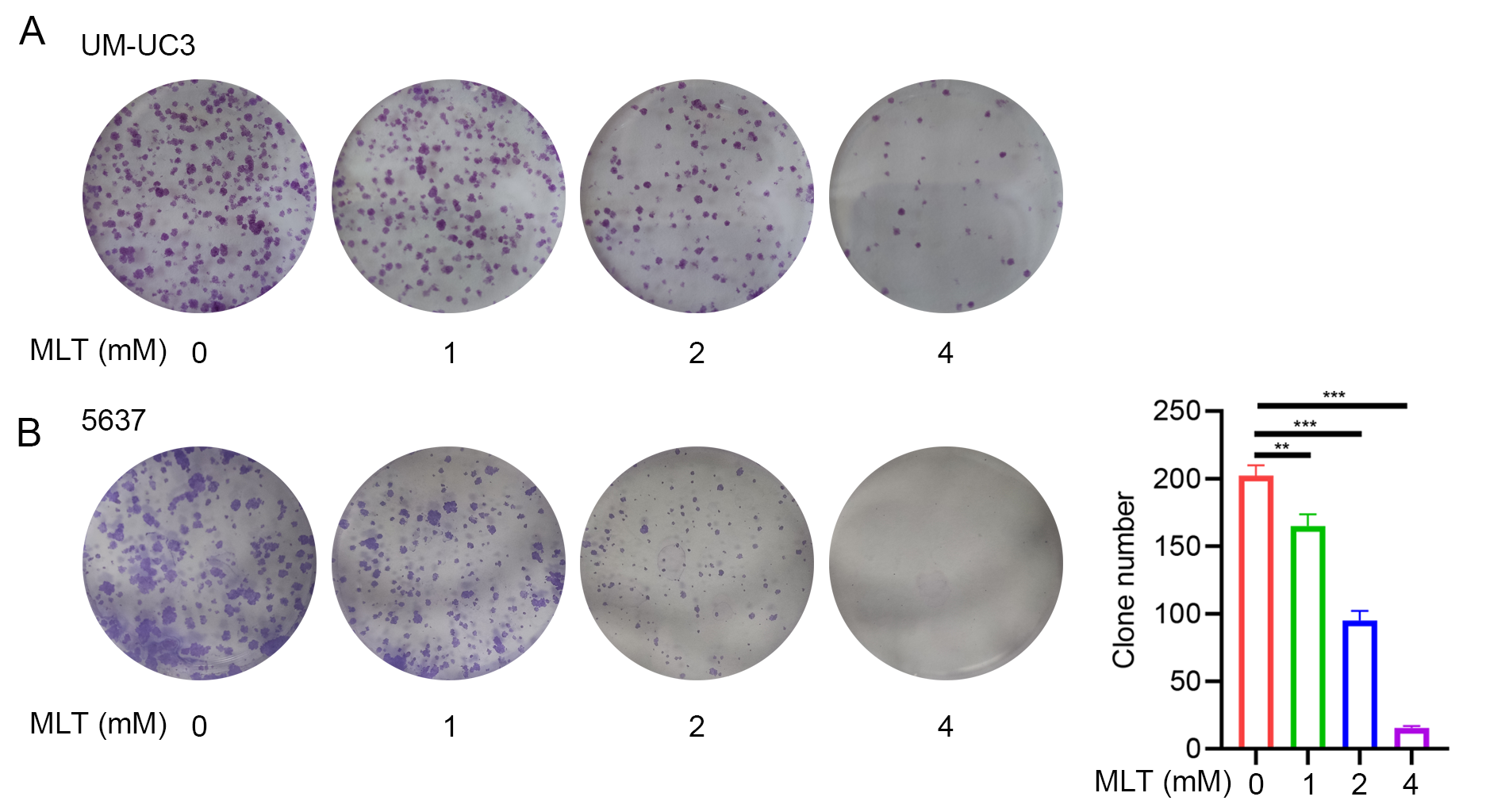
**

**Supplementary Figure S1. Melatonin exerted inhibitory effects on proliferation of BLCA cells.**

Clone formation assay of melatonin treatment on UM-UC3 cells (48 h) **(A)** and 5637 cells (24 h) **(B)** and statistical analysis (n=3). **p* < 0.05, ***p* < 0.01, ****p* < 0.001.

**
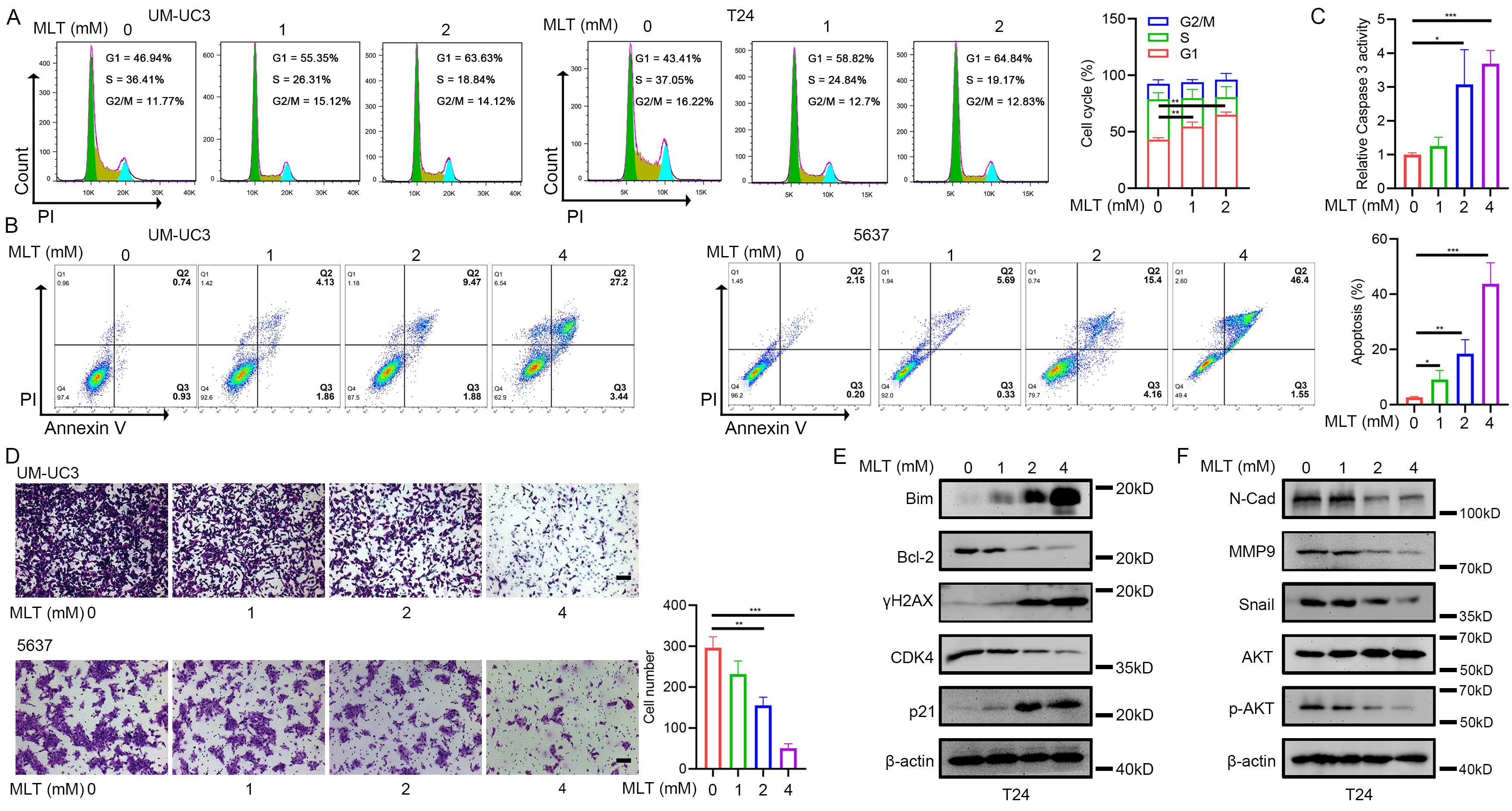
**

**Supplementary Figure S2. Melatonin exerted inhibitory effects on proliferation and metastasis of BLCA cells.**

**(A)** Flow cytometry analysis of cell cycle distribution of UM-UC3 cells and T24 cells under 24 h melatonin treatment and statistical analysis (n=3). **(B)** Apoptotic cells of UM-UC3 cells and 5637 cells under 24 h melatonin treatment and statistical analysis (n=3). **(C)** Caspase 3 activity assay of 5637 cells under 24 h melatonin treatment (n=3). **(D)** Transwell assay of UM-UC3 cells and 5637 cells under 24 h melatonin treatment and statistical analysis (n=3). Scale bar: 100 μm. **(E)** Western blot assay of apoptosis-related and cell cycle-related proteins of T24 cells after 24 h melatonin treatment. **(F)** Western blot assay of EMT-related proteins, AKT and p-AKT of T24 cells after 24 h melatonin treatment. **p* < 0.05, ***p* < 0.01, ****p* < 0.001.


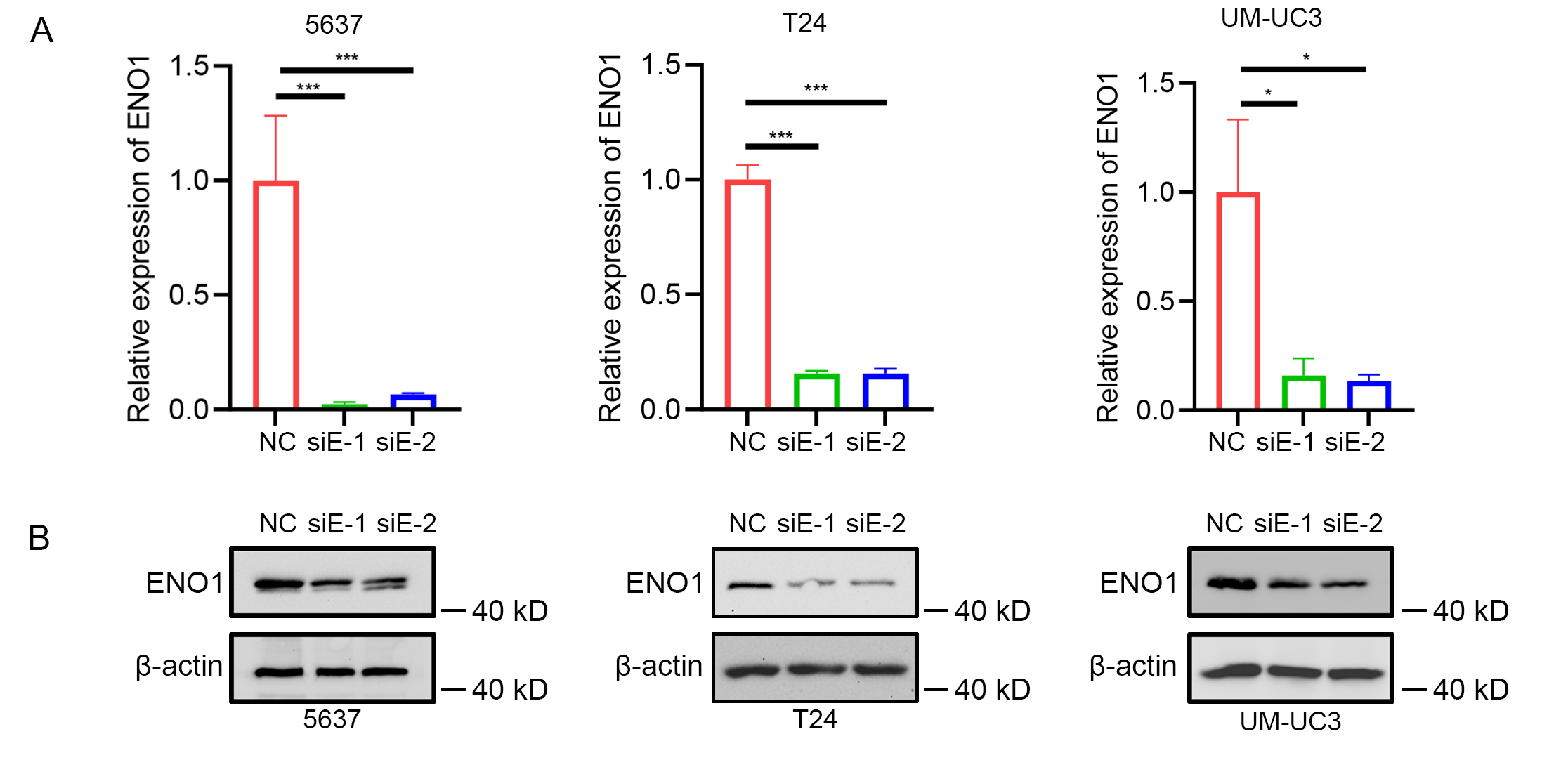


**Supplementary Figure S3. Efficiency of *ENO1* target siRNA.**

**(A)** qRT-PCR results of transfecting *ENO1* target siRNAs in BLCA cells (n=3). **(B)** Western blot results of transfecting *ENO1* target siRNAs in BLCA cells. **p* < 0.05, ***p* < 0.01, ****p* < 0.001.


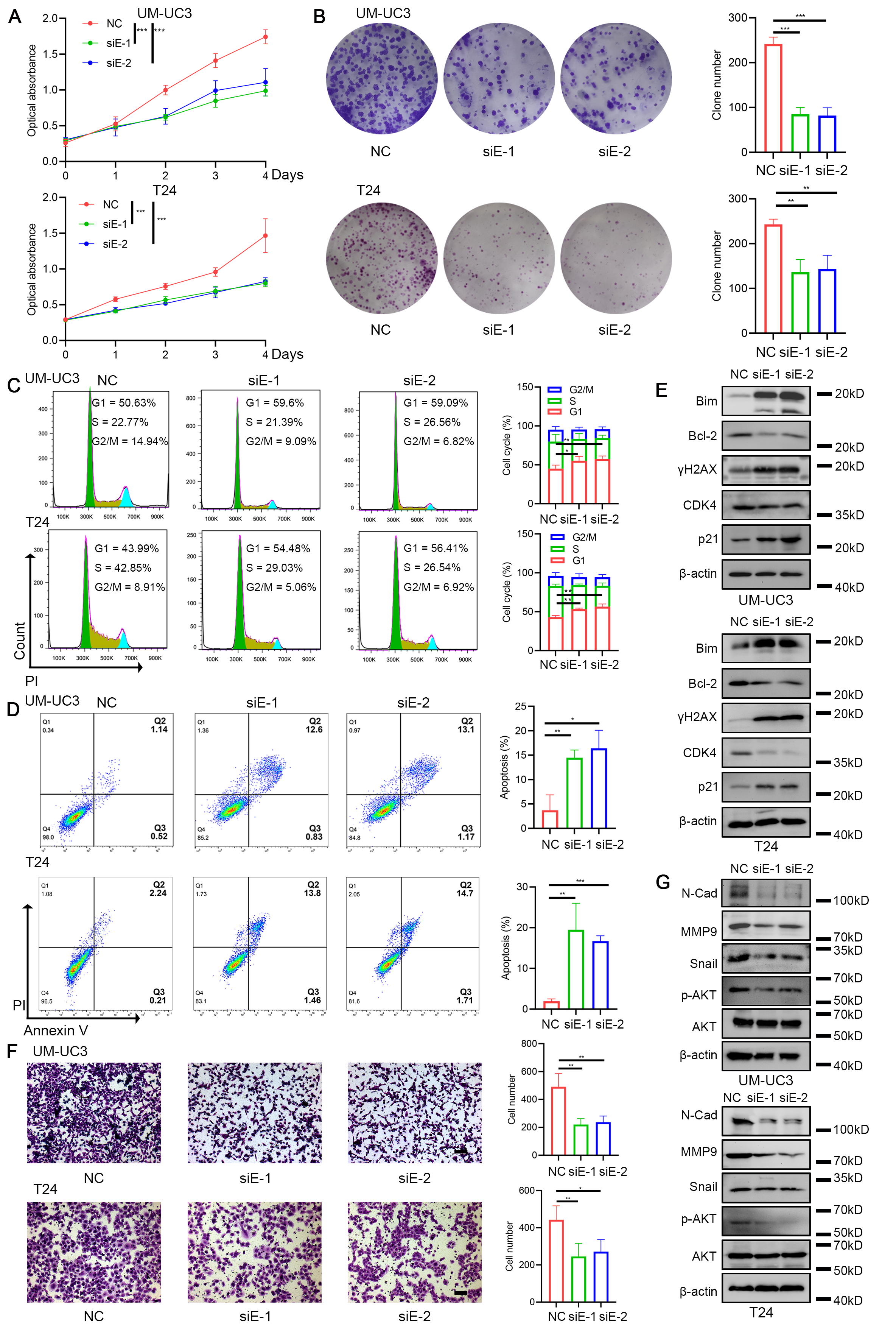


**Supplementary Figure S4. Silencing of *ENO1* inhibited proliferation and metastasis of BLCA cells.**

**(A)** MTT assay of UM-UC3 cells and T24 cells after silencing *ENO1* (n=3). **(B)** Clone formation assay of UM-UC3 cells and T24 cells after silencing *ENO1* and statistical analysis (n=3). **(C)** Flow cytometry analysis of cell cycle distribution of UM-UC3 cells and T24 cells after silencing *ENO1* and statistical analysis (n=3). **(D)** Flow cytometry analysis of apoptotic cells of UM-UC3 cells and T24 cells after silencing *ENO1* and statistical analysis (n=3). **(E)** Western blot assay of apoptosis-related and cell cycle-related proteins of UM-UC3 cells and T24 cells after silencing *ENO1*. **(F)** Transwell assay of UM-UC3 cells and T24 cells after silencing *ENO1* and statistical analysis. Scale bar: 100 μm (n=3). **(G)** Western blot assay of EMT-related proteins, AKT and p-AKT of UM-UC3 cells and T24 cells after silencing *ENO1*. **p* < 0.05, ***p* < 0.01, ****p* < 0.001.


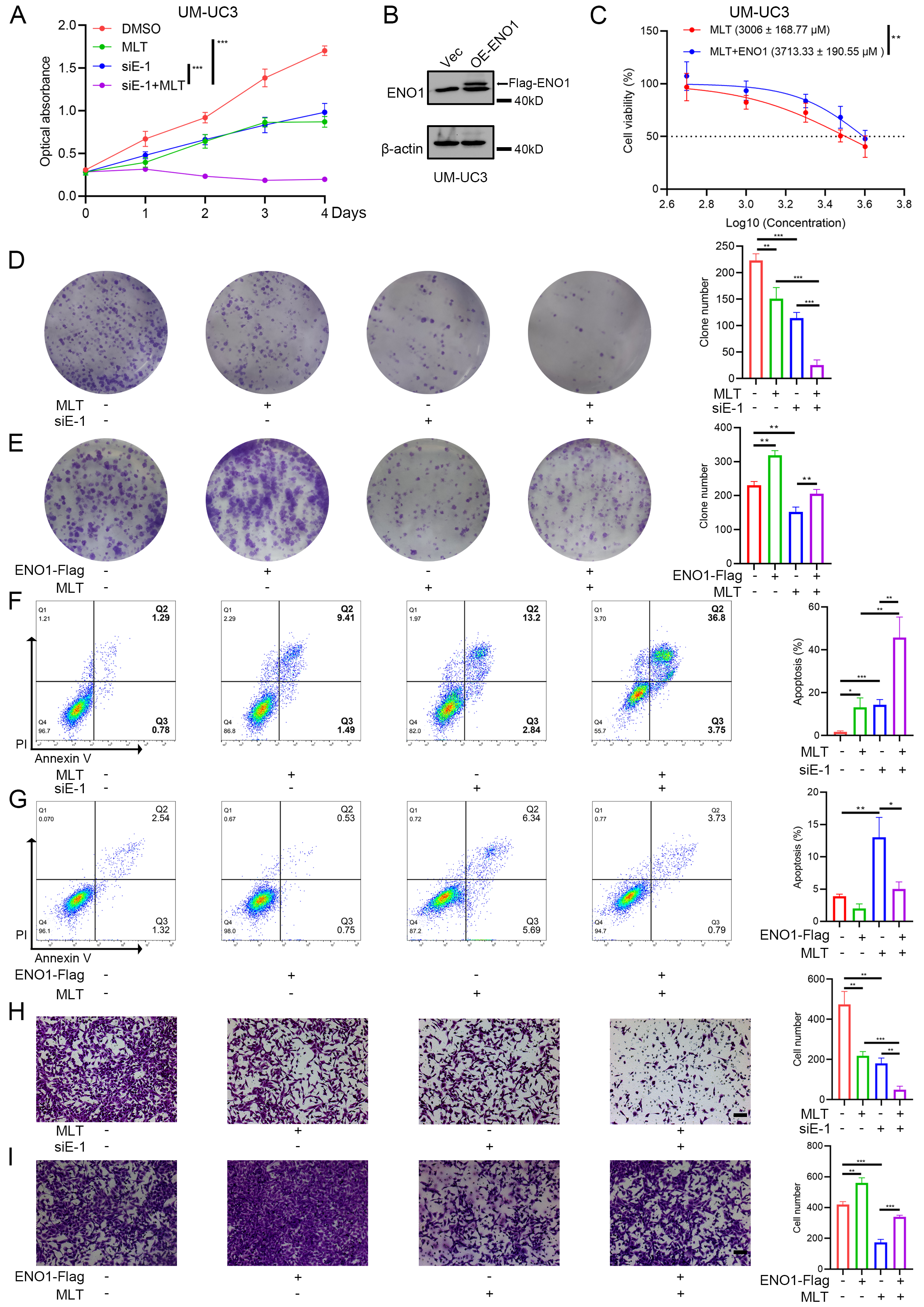


**Supplementary Figure S5. *ENO1* mediated the inhibitory effect of melatonin on BLCA cells.**

**(A)** MTT assay of UM-UC3 cells treated with melatonin (2 mM) after silencing *ENO1* (n=3). **(B)** Western blot results of over-expressing Flag-ENO1 plasmid in UM-UC3 BLCA cells. **(C)** IC50 value of UM-UC3 cells treated with melatonin after over-expressing ENO1 (n=3). **(D)** Clone formation assay of UM-UC3 cells treated with 48 h melatonin (2 mM) after silencing *ENO1* and statistical analysis (n=3). **(E)** Clone formation assay of UM-UC3 cells treated with 48 h melatonin (2 mM) after over-expressing ENO1 and statistical analysis (n=3). **(F)** Apoptotic cells of UM-UC3 cells were treated with 24 h melatonin (2 mM) after silencing *ENO1* and statistical analysis (n=3). **(G)** Apoptotic cells of UM-UC3 cells were treated with 24 h melatonin (2 mM) after over-expressing ENO1 and statistical analysis (n=3). **(H)** Transwell assay of UM-UC3 cells treated with 24 h melatonin (2 mM) after silencing *ENO1* and statistical analysis (n=3). Scale bar: 100 μm. **(I)** Transwell assay of UM-UC3 cells treated with 24 h melatonin (2 mM) after over-expressing ENO1 and statistical analysis (n=3). Scale bar: 100 μm. **p* < 0.05, ***p* < 0.01, ****p* < 0.001.


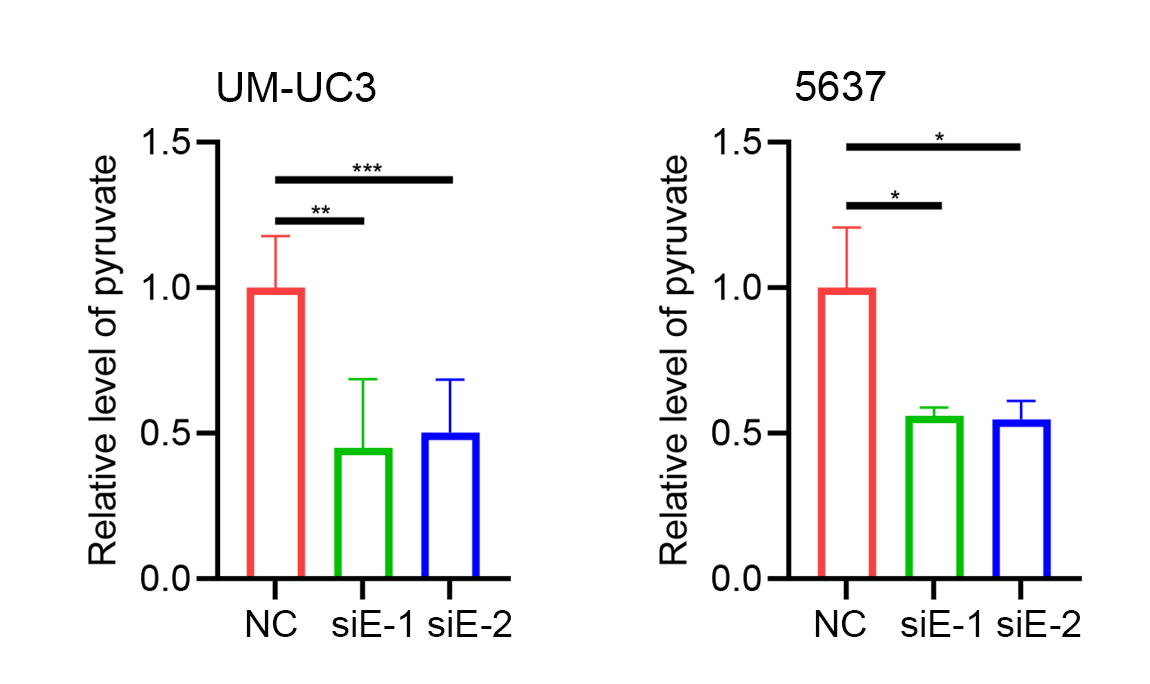


**Supplementary Figure S6. Silencing *ENO1* reduced cellular pyruvate level in BLCA cells.**

**(A)** Detection of cellular pyruvate level after silencing *ENO1* in UM-UC3 cells (n=3). **(B)** Detection of cellular pyruvate level after silencing *ENO1* in 5637 cells (n=3). **p* < 0.05, ***p* < 0.01, ****p* < 0.001.


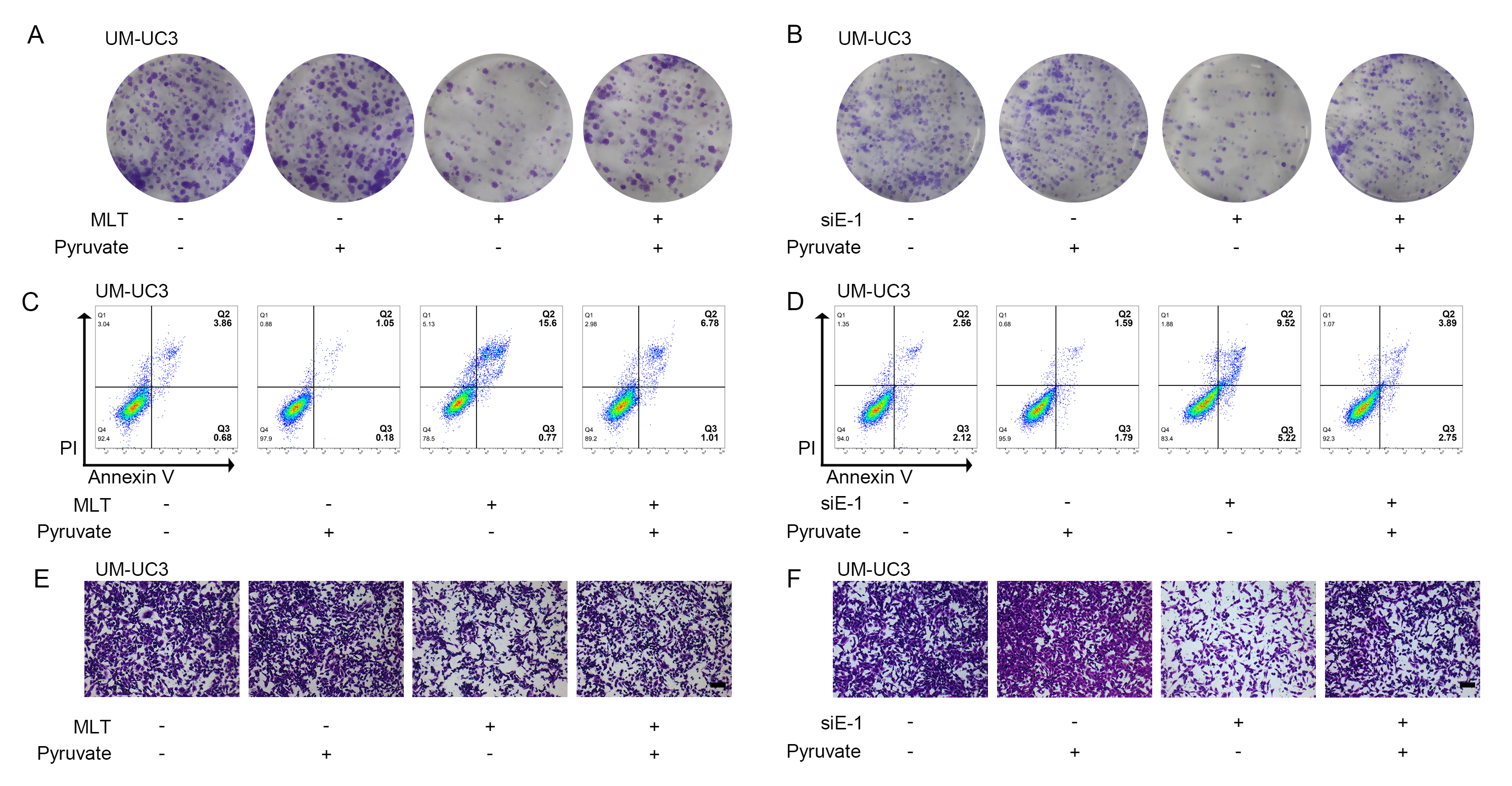


**Supplementary Figure S7. Supplement of exogenous pyruvate could reverse the inhibitory effect of melatonin treatment or silencing *ENO1* on BLCA cells.**

**(A)** Clone formation assay of UM-UC3 cells under 48 h melatonin (2 mM) treatment and exogenous pyruvate (3 mM) supplement. **(B)** Clone formation assay of UM-UC3 cells after silencing *ENO1* with exogenous pyruvate (3 mM) supplement. **(C)** Apoptotic cells of UM-UC3 cells after 24 h melatonin (2 mM) treatment with exogenous pyruvate (3 mM) supplement. **(D)** Apoptotic cells of UM-UC3 cells after silencing *ENO1* with exogenous pyruvate (3 mM) supplement. **(E)** Transwell assay of UM-UC3 cells after 24 h melatonin (2 mM) treatment with exogenous pyruvate (3 mM) supplement. Scale bar: 100 μm. **(F)** Transwell assay of UM-UC3 cells after silencing *ENO1* with exogenous pyruvate (3 mM) supplement. Scale bar: 100 μm.


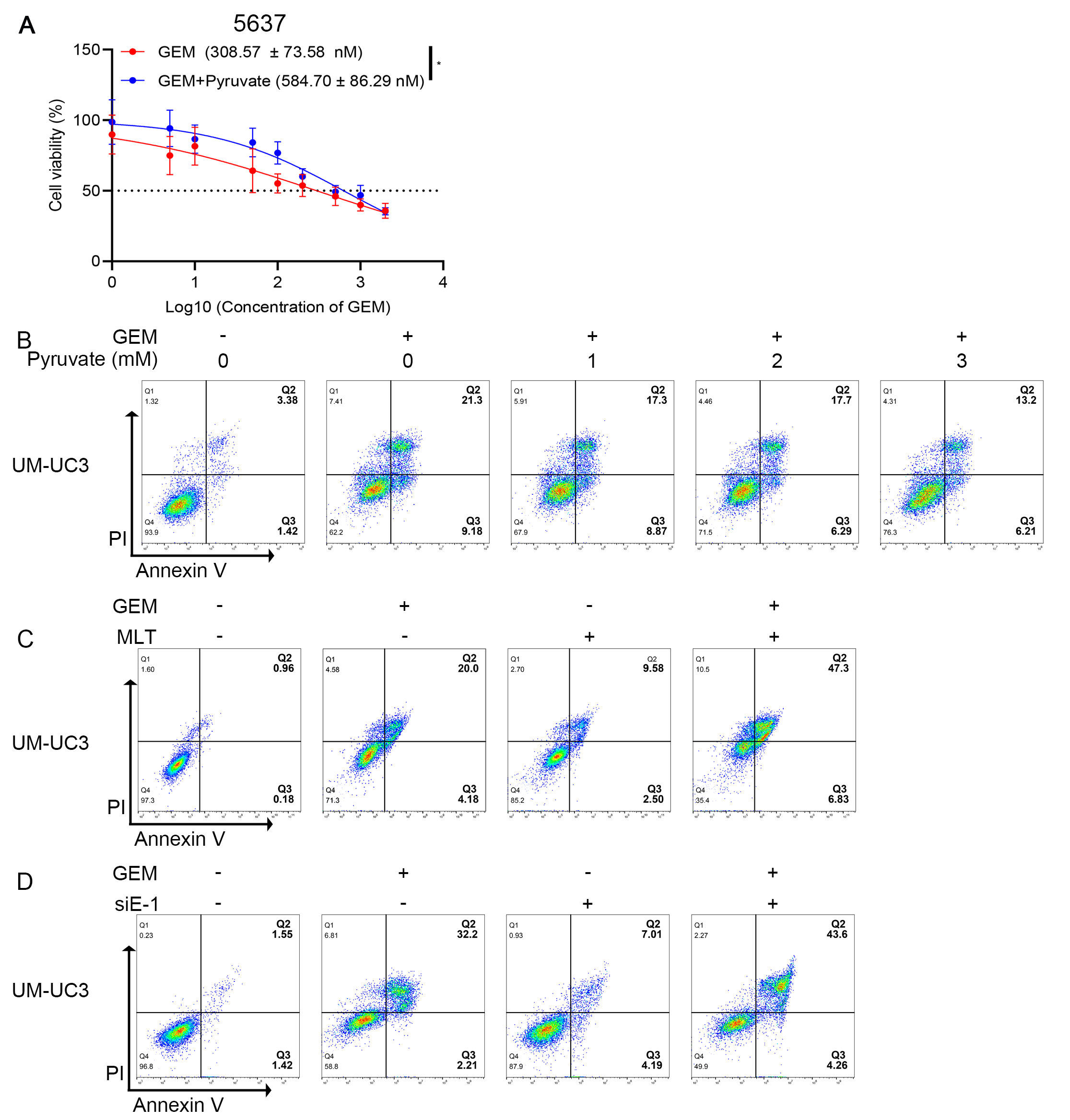


**Supplementary Figure S8. Melatonin treatment or silencing *ENO1* could promote the cytotoxic effect of gemcitabine.**

**(A)** IC50 value of 48 h gemcitabine treatment and gemcitabine treatment combined with pyruvate (3 mM) supplement on 5637 cells (n=3). **(B)** Apoptotic cells of UM-UC3 cells after 48 h gemcitabine (0.5 μM) treatment with exogenous pyruvate (3 mM) supplement. **(C)** Apoptotic cells of UM-UC3 cells with 48 h gemcitabine (0.5 μM) treatment and 24 h melatonin (2 mM) treatment. **(D)** Apoptotic cells of UM-UC3 cells with 48 h gemcitabine (0.5 μM) treatment and silencing *ENO1*. **p* < 0.05, ***p* < 0.01, ****p* < 0.001.


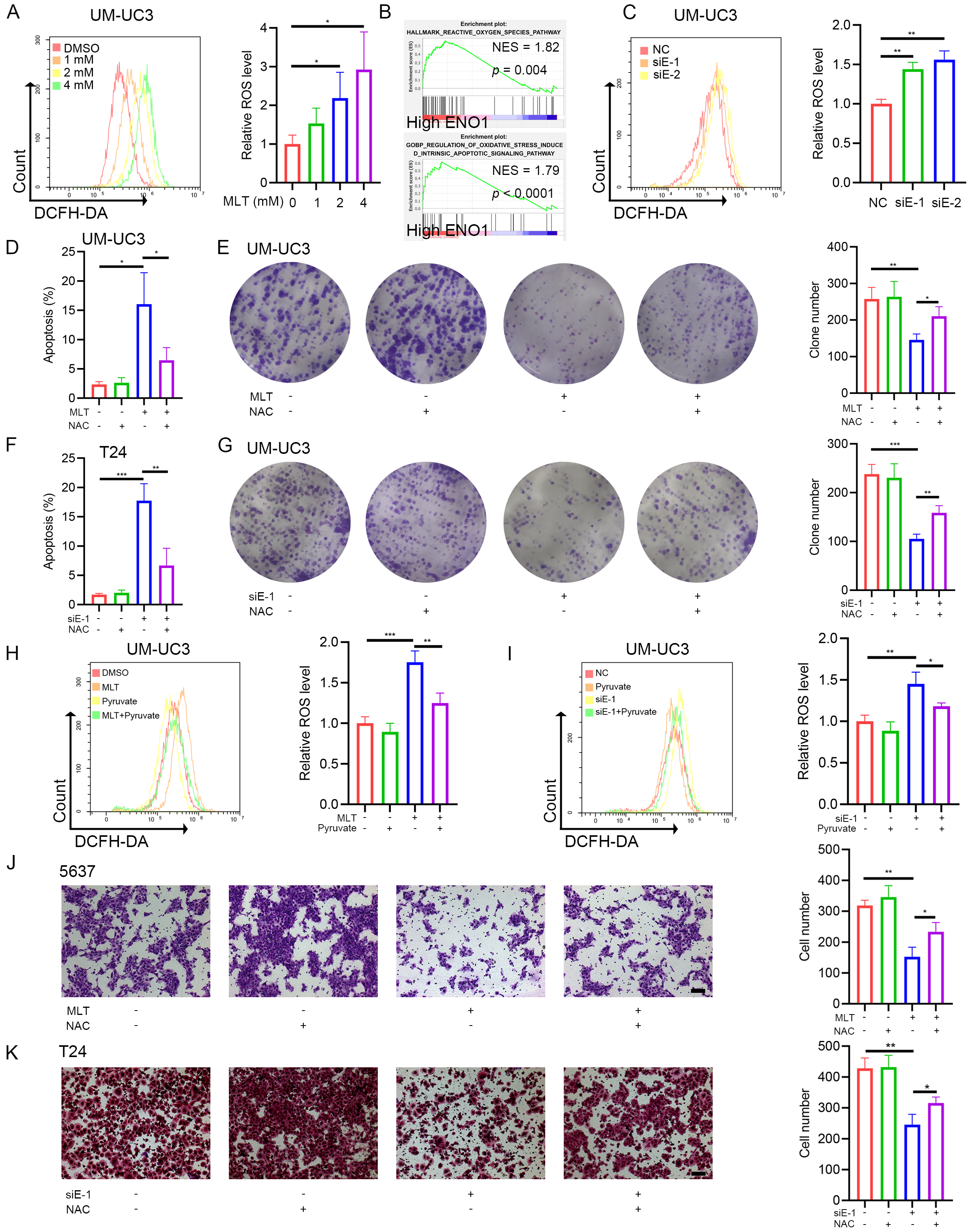


**Supplementary Figure S9. ROS mediated the inhibitory effect of melatonin or silencing *ENO1* on BLCA cells.**

**(A)** Alteration of ROS level in UM-UC3 cells after 24 h melatonin treatment and statistical analysis (n=3). **(B)** GSEA results of CCLE database based on *ENO1* expression level in BLCA cell lines. **(C)** Alteration of ROS level in UM-UC3 cells after silencing *ENO1* and statistical analysis (n=3). **(D)** Apoptotic cells of UM-UC3 cells after 24 h melatonin (2 mM) treatment with NAC (5 mM) supplement (n=3). **(E)** Clone formation assay of under 48 h melatonin (2 mM) treatment with NAC (5 mM) supplement in UM-UC3 cells and statistical analysis (n=3). **(F)** Apoptotic cells of T24 cells after silencing ENO1 with NAC (5 mM) supplement (n=3). **(G)** Clone formation assay of silencing *ENO1* with NAC (5 mM) supplement in UM-UC3 cells and statistical analysis (n=3). **(H)** Alteration of ROS level in UM-UC3 cells after 24 h melatonin treatment with exogenous pyruvate (3 mM) supplement and statistical analysis (n=3). **(I)** Alteration of ROS level in UM-UC3 cells after silencing of *ENO1* with exogenous pyruvate (3 mM) supplement and statistical analysis (n=3). **(J)** Transwell assay of 5637 cells after 24 h melatonin (2 mM) treatment with NAC (5 mM) supplement and statistical analysis (n=3). Scale bar: 100 μm. **(K)** Transwell assay of T24 cells after silencing *ENO1* with NAC (5 mM) supplement and statistical analysis (n=3). Scale bar: 100 μm. **p* < 0.05, ***p* < 0.01, ****p* < 0.001.


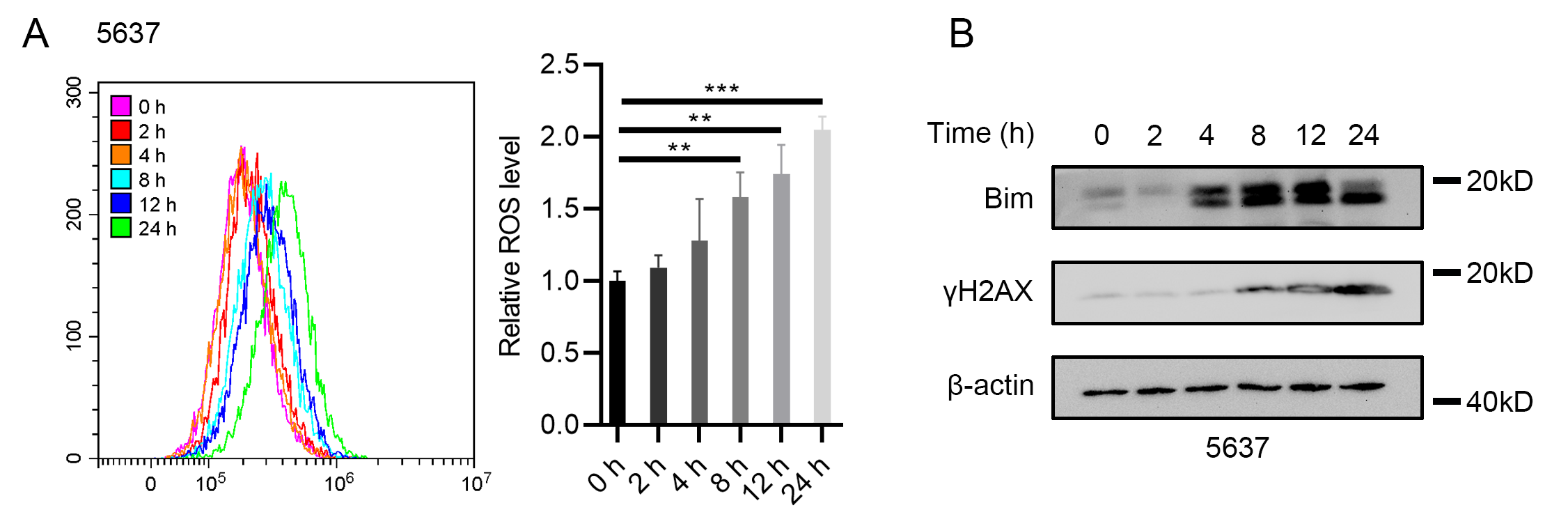


**Supplementary Figure S10. Melatonin treatment caused ROS accumulation and induced apoptosis signaling pathway in a time-dependent manner.**

**(A)** Detection of cellular ROS level after melatonin (2 mM) treatment at the annotated time point in 5637 BLCA cells and statistical analysis (n=3). **(B)** Western blot of Bim and γH2AX level after 24 h melatonin (2 mM) treatment at the annotated time point in 5637 BLCA cells. **p* < 0.05, ***p* < 0.01, ****p* < 0.001.


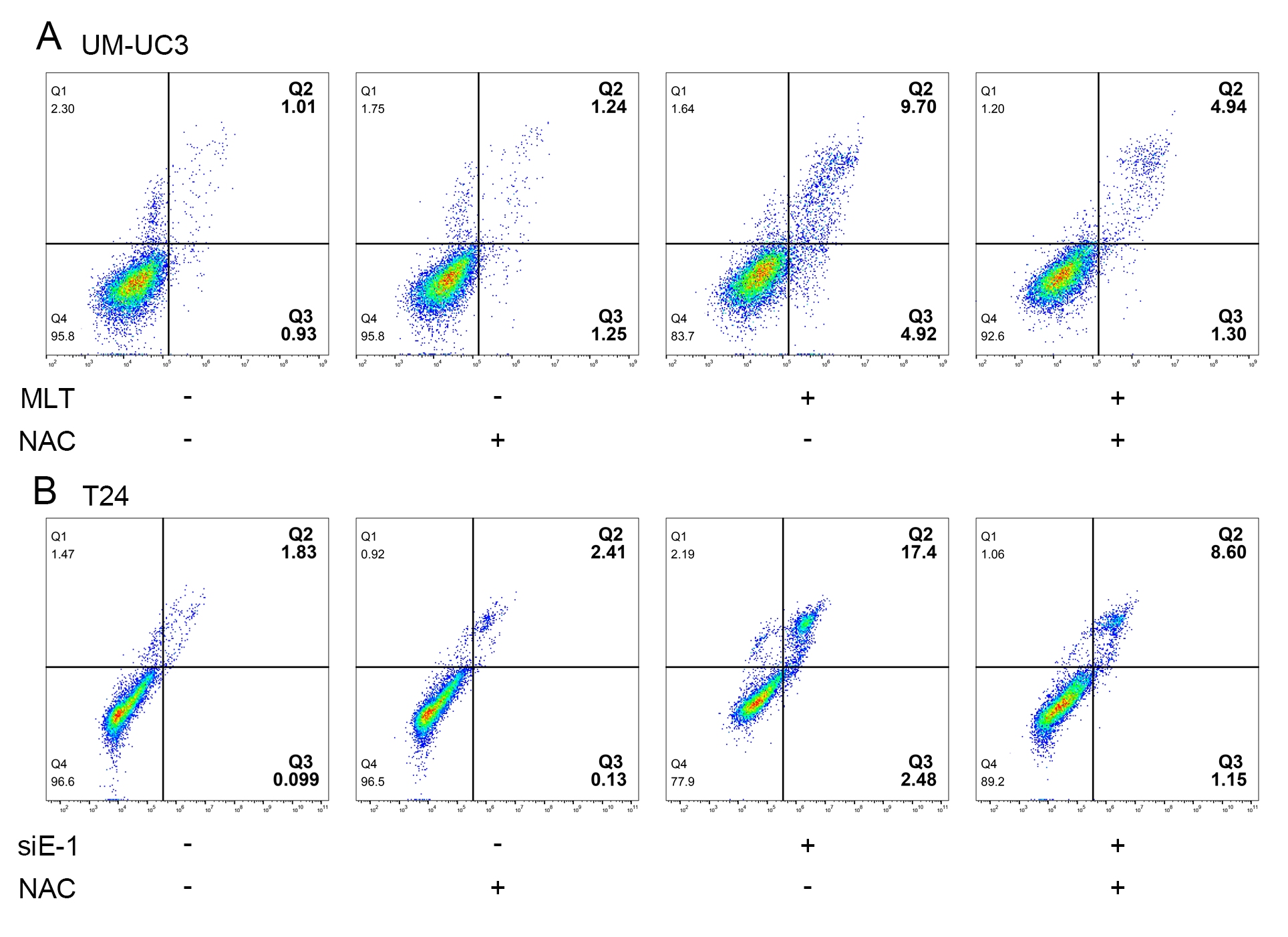


**Supplementary Figure S11. ROS mediated the inhibitory effect of melatonin or silencing *ENO1* on BLCA cells.**

**(A)** Apoptotic cells of UM-UC3 cells after 24 h melatonin (2 mM) treatment with NAC (5 mM) supplement. **(B)** Apoptotic cells of T24 cells after silencing *ENO1* with NAC (5 mM) supplement, negative control siRNA was added in “-” of siE-1 group.


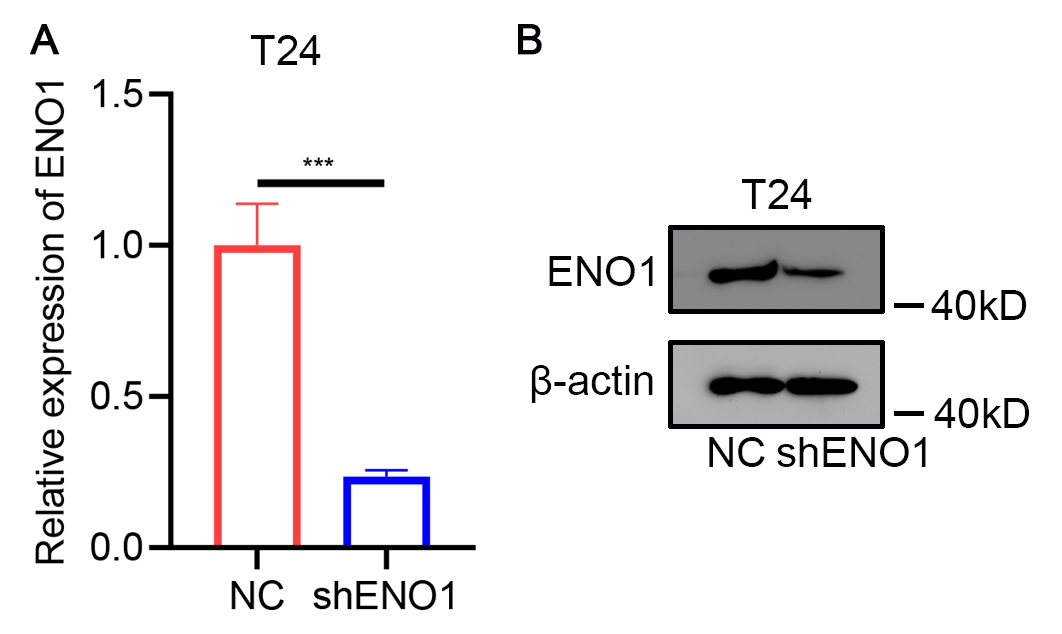


**Supplementary Figure S12. Melatonin treatment or *ENO1* silencing could suppress BLCA growth *in vivo.***

**(A)** qRT-PCR results of transfecting LV-*shENO1* in T24 cells (n=4). **(B)** Western blot analysis of transfecting LV-*shENO1* in T24 cells. **p* < 0.05, ***p* < 0.01, ****p* < 0.001.


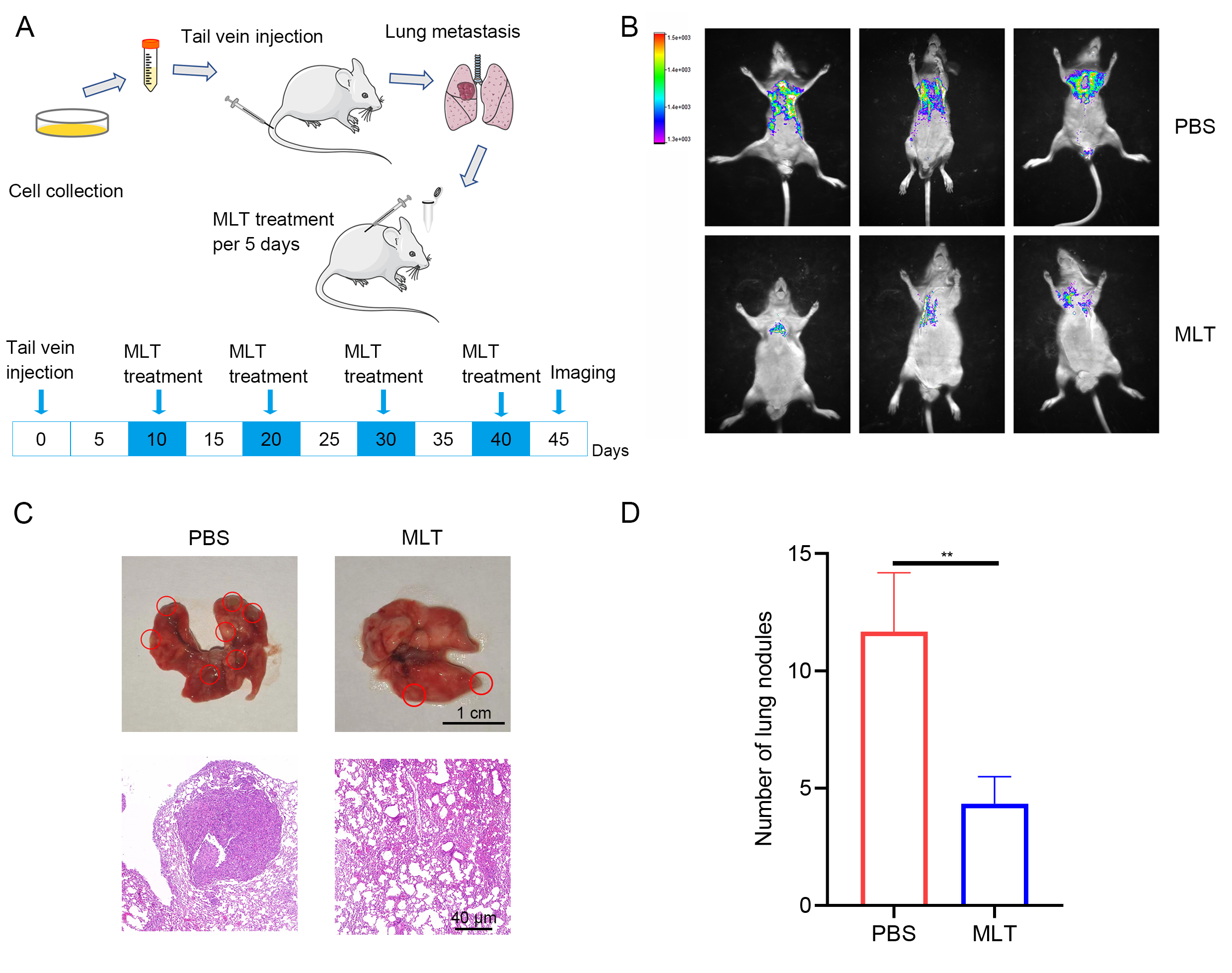


**Supplementary Figure S13. Melatonin treatment or *ENO1* silencing could suppress BLCA metastasis *in vivo.***

**(A)** Pattern of lung metastasis model construction and drug treatment. **(B)** *In vivo* imaging of lung-metastasis model. **(C)** Representative pictures of dissected lungs and H&E staining results. Scale bar: 1 cm or 40 μm. **(D)** Statistical analysis of lung nodules (n=3). **p* < 0.05, ***p* < 0.01, ****p* < 0.001.


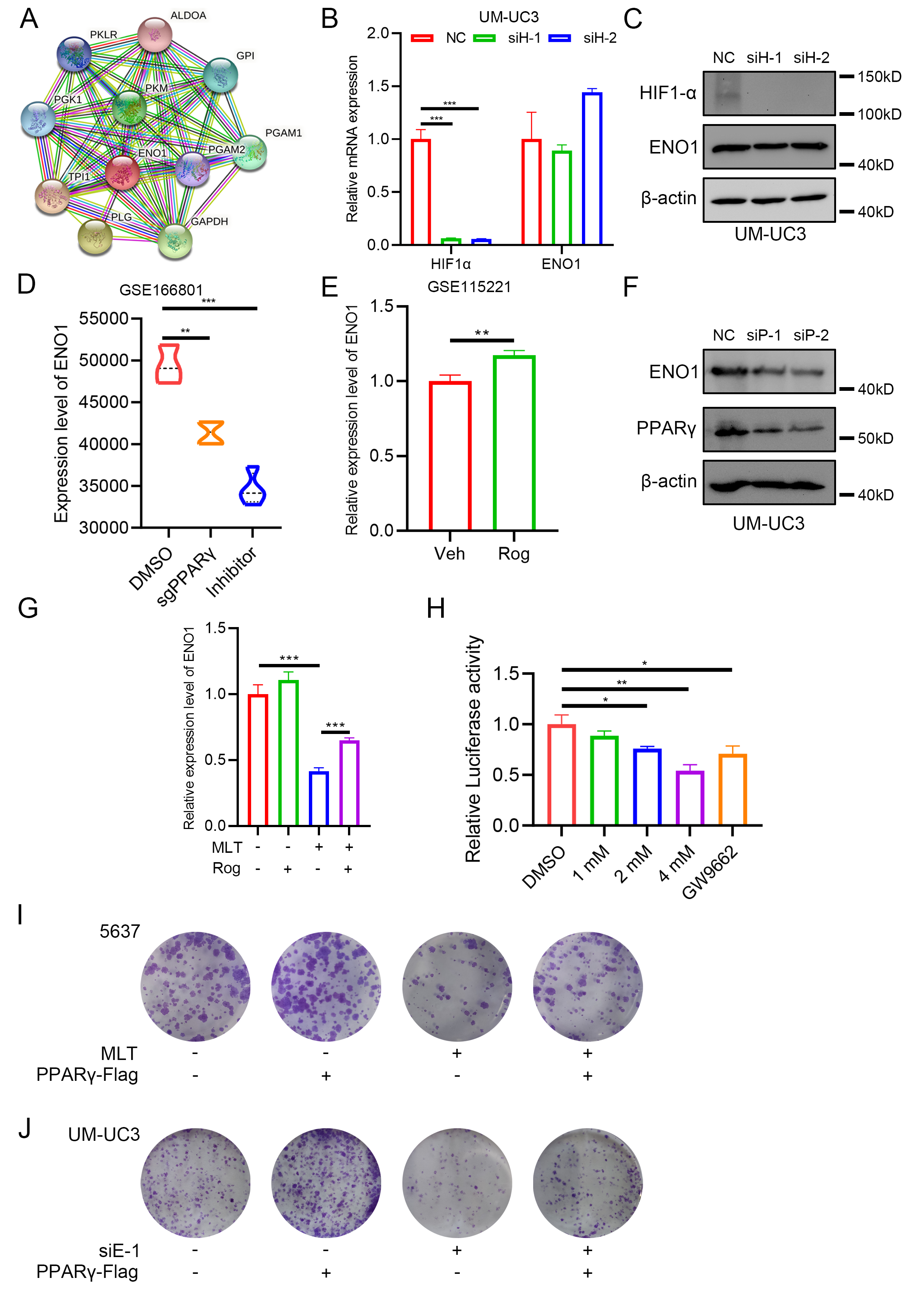


**Supplementary Figure S14. PPARγ mediated the down-regulation of *ENO1* by melatonin.**

**(A)** Interaction network of ENO1 from STRING database. **(B)** qRT-PCR results of *ENO1* and *HIF1α* mRNA level after silencing *HIF1α* in UM-UC3 cells (n=4). **(C)** Western blot results of ENO1 and HIF1α protein level after silencing *HIF1α* in UM-UC3 cells. **(D)** Expression status of *ENO1* in GSE166801. **(E)** Expression status of *ENO1* in GSE115221. **(F)** Western blot results of ENO1 protein level after silencing *PPAR****γ*** in UM-UC3 cells. **(G)** qRT-PCR results of *ENO1* mRNA level after 36 h melatonin (4 mM) treatment and 24 h Rosiglitazone (5 μM) treatment (n=4). **(H)** Dual luciferase reporter assay of ENO1 promoter activity (n=3). **(I)** Clone formation assay of 5637 cells with PPAR**γ** overexpression and 24 h melatonin (2 mM) treatment. **(J)** Clone formation assay of UM-UC3 cells with PPARγ overexpression and silencing *ENO1*. **p* < 0.05, ***p* < 0.01, ****p* < 0.001.


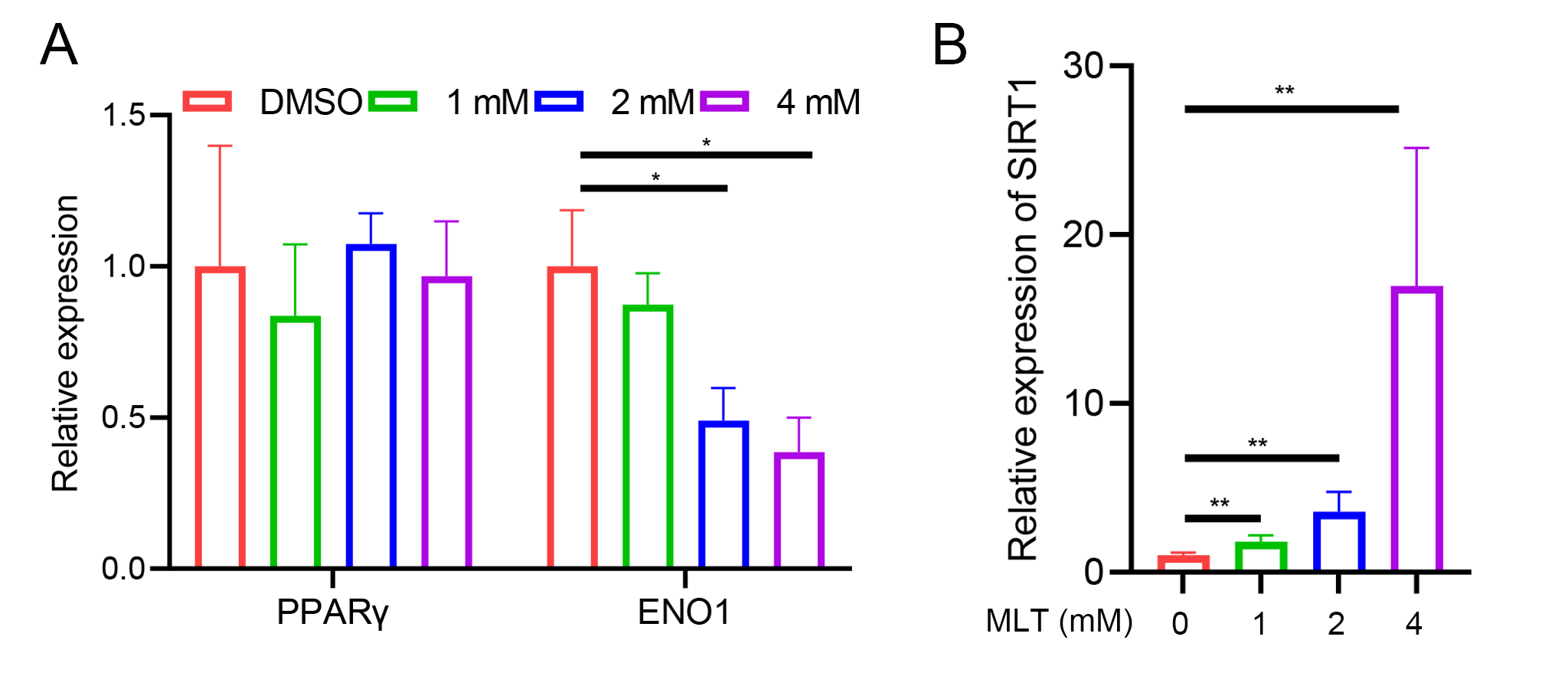


**Supplementary Figure S15. PPARγ mediated the down-regulation of *ENO1* by melatonin.**

**(A)** qRT-PCR analysis of *PPARγ* and *ENO1* mRNA level in UM-UC3 cells under melatonin treatment (n=4). **(B)** qRT-PCR analysis of *SIRT1* mRNA level in UM-UC3 cells under melatonin treatment (n=4). **p* < 0.05, ***p* < 0.01, ****p* < 0.001.
