# Supplementary material for: Melatonin inhibits bladder tumorigenesis by suppressing PPARγ/ENO1-mediated glycolysis
Source: Cell Death Dis. 2023 Apr 6;14(4):246. doi: 10.1038/s41419-023-05770-8 (PMC10079981; doi:10.1038/s41419-023-05770-8)
Supplement: Supplementary file 2 — Supplementary Tables S1-S4 [file 41419_2023_5770_MOESM2_ESM.docx]

**Supplementary Tables S1-S4**

**Supplementary Table S1. Sequences of siRNAs used in this study.**

| **Target** | **Sequence (5′‐3′)** |
| --- | --- |
| siENO1-1 (siE-1) | CCCAGUGGUGUCUAUCGAATT |
| siENO1-2 (siE-2) | GCAUUGGAGCAGAGGUUUATT |
| siPPARγ-1 (siP-1) | GGUUGCAGAUUACAAGUAUTT |
| siPPARγ-2 (siP-2) | GCGGAGAUCUCCAGUGAUATT |
| siHIF1α-1 (siH-1) | GCGAAGUAAAGAAUCUGAAGU |
| siHIF1α-2 (siH-2) | CGAUGGAAGCACUAGACAAAG |
| NC | UUCUCCGAACGUGUCACGUTT |

**Supplementary Table S2. Primers for qRT-PCR used in this study.**

| **Gene** | **Forward primer (5′‐3′)** | **Reverse primer (5′‐3′)** |
| --- | --- | --- |
| ENO1 | GTTCACAGCCAGTGCAGGAA | GGAGGCAGTTGCAGGACTTC |
| β-actin | CATGTACGTTGCTATCCAGGC | CTCCTTAATGTCACGCACGAT |
| PPARγ | ATGACAGACCTCAGACAGATTG | AATGTTGGCAGTGGCTCAG |
| SIRT1 | TAGCCTTGTCAGATAAGGAAGGA | ACAGCTTCACAGTCAACTTTGT |
| HIF1α | TCCATGTGACCATGAGGAAA | AAGCTTCGCTGTGTGTTTTG |
| ENO1-ChIP-P1 | ACTCCTTCCGTATTCCACGG | CTGGAATTTCACACAGACCCG |
| ENO1-ChIP-P2 | AAGTGAACCTTCCTTTGGCTGT | AAGGTGACCCTGTCCCTTTCT |
| ENO1-ChIP-P3 | GTCAGCAAGGTCGAGGGC | GCTCCGTCACGTACTCCG |

**Supplementary Table S3. Primary antibodies used in this study.**

| **Antigens** | **Host** | **IHC** | **WB** | **ChIP** | **Supplier** |
| --- | --- | --- | --- | --- | --- |
| β-actin | Mouse |  | 1:1000 |  | Santa Cruz, sc‐47778 |
| ENO1 | Rabbit |  | 1:1000 |  | Abcam, ab227978 |
| p21 | Rabbit |  | 1:1000 |  | CST, 2947 |
| CDK4 | Rabbit |  | 1:1000 |  | CST, 12790 |
| MMP9 | Rabbit |  | 1:1000 |  | CST, 13667 |
| N-Cad | Rabbit |  | 1:1000 |  | CST, 13116 |
| PPARγ | Rabbit |  | 1:1000 | 1:100 | CST, 2435 |
| Snail | Rabbit |  | 1:1000 |  | CST, 3879 |
| γH2AX | Rabbit |  | 1:1000 |  | Abcam, ab81299 |
| AKT | Rabbit |  | 1:1000 |  | CST, 4691 |
| p-AKT (Ser473) | Rabbit |  | 1:1000 |  | CST, 4060 |
| Bim | Rabbit |  | 1:1000 |  | CST, 2933 |
| HIF1α | Rabbit |  | 1:1000 |  | Proteintech, 20960-1-AP |
| Bcl-2 | Mouse |  | 1:500 |  | CST, 15071 |
| Ki67 | Rabbit | 1:100 |  |  | Novus, NBP2‐19012 |

**Supplementary Table S4. Function analysis of *ENO1* via STRING online database.**

| **Term ID** | **Term description** | **Strength** | **FDR** |
| --- | --- | --- | --- |
| hsa00010 | Glycolysis / Gluconeogenesis | 2.44 | <0.001^***^ |
| GO:0061621 | Canonical glycolysis | 2.82 | <0.001^***^ |
| GO:0042866 | Pyruvate biosynthetic process | 2.77 | 0.0015^**^ |
| GO:1903580 | Positive regulation of ATP metabolic process | 1.88 | 0.0403^*^ |
| WP4629 | Aerobic glycolysis | 3.07 | <0.001^***^ |
| WP2456 | HIF1α and PPARγ regulation of glycolysis | 2.65 | 0.0012^**^ |

* *p* < 0.05, ** *p* < 0.01, ****p* < 0.001.
