# Supplementary material for: Melatonin inhibits bladder tumorigenesis by suppressing PPARγ/ENO1-mediated glycolysis
Source: Cell Death Dis. 2023 Apr 6;14(4):246. doi: 10.1038/s41419-023-05770-8 (PMC10079981; doi:10.1038/s41419-023-05770-8)
Supplement: Supplementary file 3 — Related file. Original Western blots [file 41419_2023_5770_MOESM3_ESM.pdf]

Unedited gel in Figure 1G

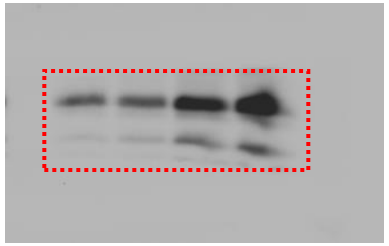

Bim

MLT (mM) 0 1 2 4

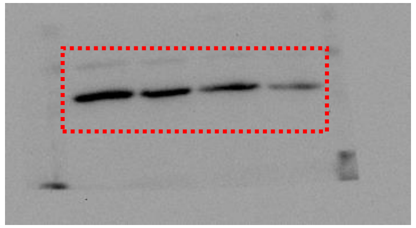

CDK4

MLT (mM) 0 1 2 4

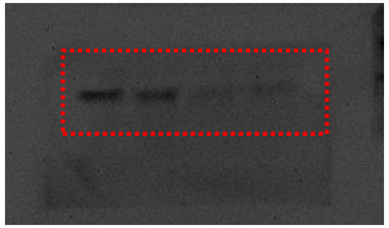

Bcl-2

MLT (mM) 0 1 2 4

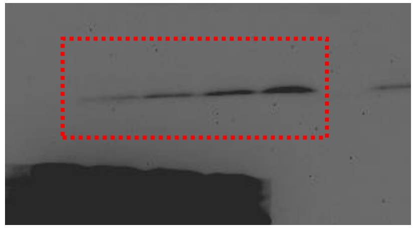

p21

MLT (mM) 0 1 2 4

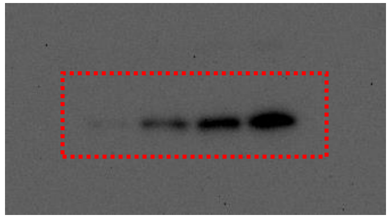

γH2AX

MLT (mM) 0 1 2 4

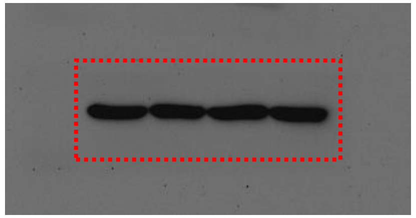

β-actin

MLT (mM) 0 1 2 4

Unedited gel in Figure 1I

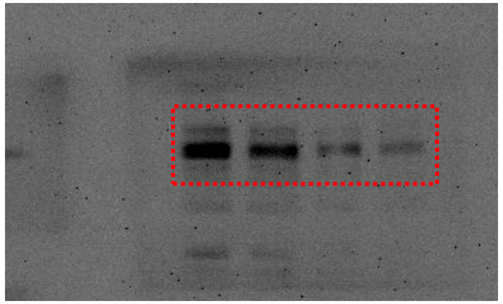

N-Cad

MLT (mM) 0 1 2 4

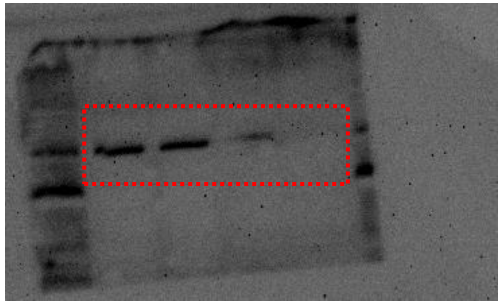

MMP9

MLT (mM) 0 1 2 4

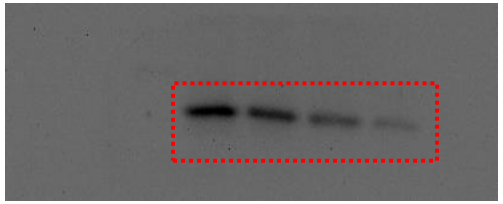

Snail

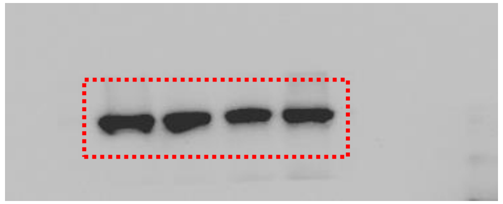

AKT

MLT (mM) 0 1 2 4

MLT (mM) 0 1 2 4

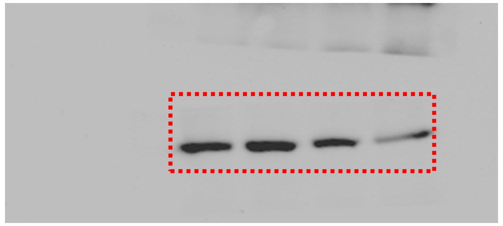

p-AKT

MLT (mM) 0 1 2 4

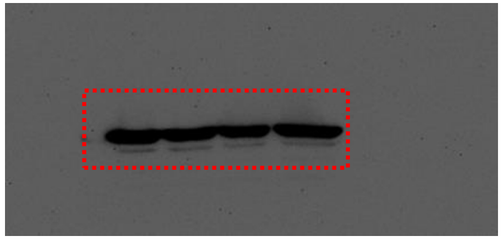

β-actin

MLT (mM) 0 1 2 4

Unedited gel in Figure 2J

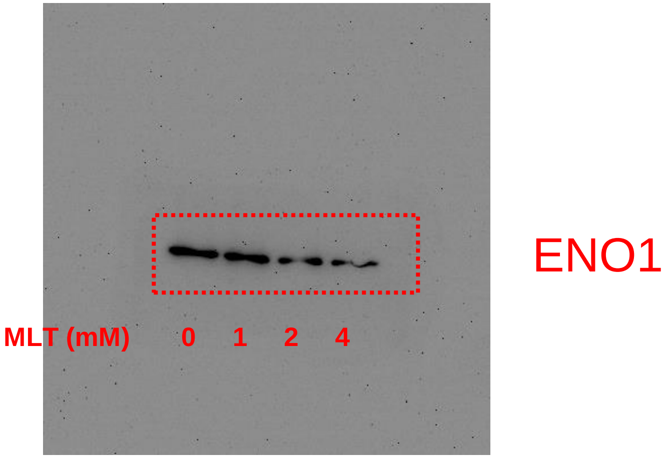

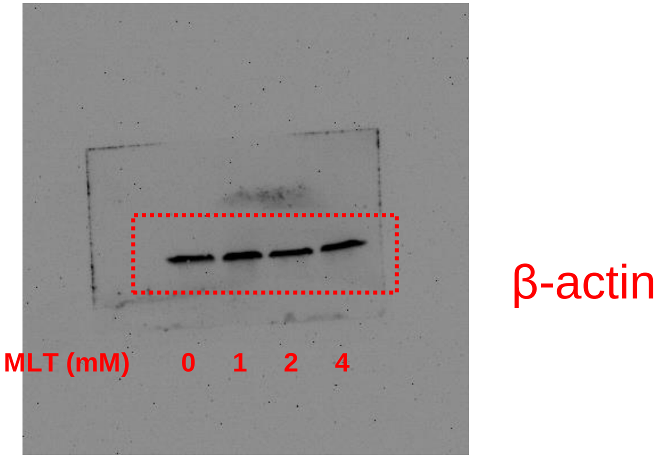

Unedited gel in Figure 3D

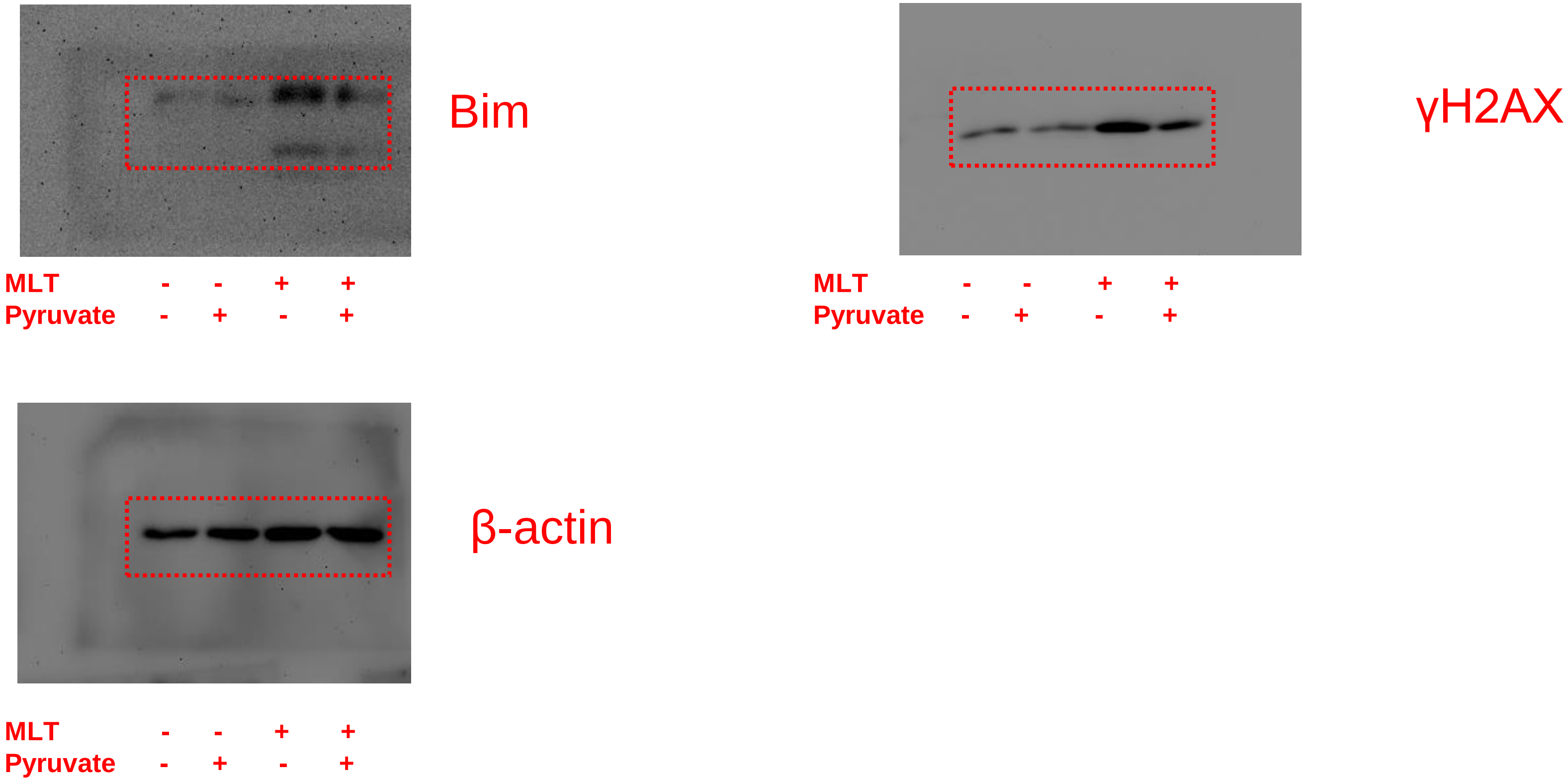

Unedited gel in Figure 3H

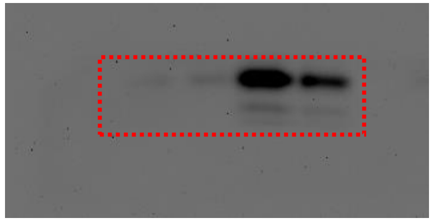

Bim

siE-1 - - + +
Pyruvate - + - +

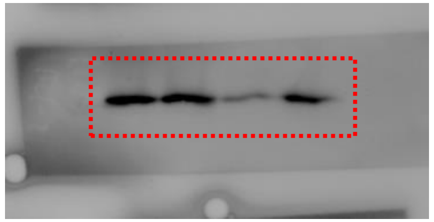

Bcl-2

siE-1 - - + +
Pyruvate - + - +

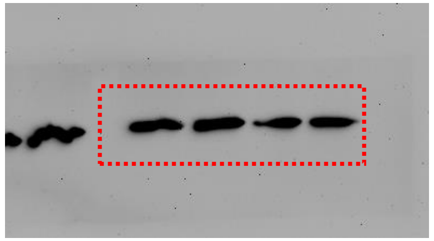

β-actin

siE-1 - - + +
Pyruvate - + - +

Unedited gel in Figure 3J

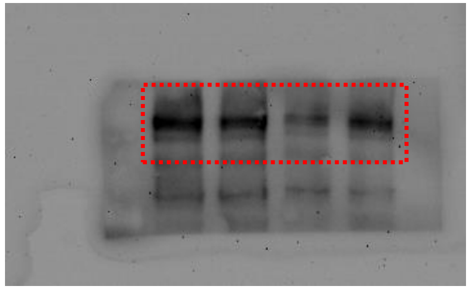

N-Cad

MLT	-	-	+	+
Pyruvate	-	+	-	+

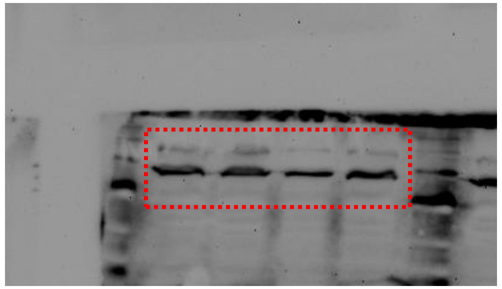

MMP9

MLT	-	-	+	+
Pyruvate	-	+	-	+

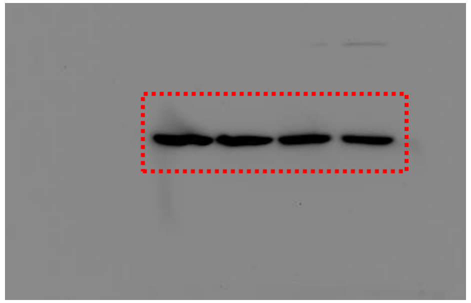

β-actin

MLT	-	-	+	+
Pyruvate	-	+	-	+

Unedited gel in Figure 3L

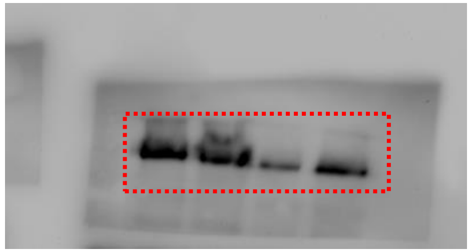

N-Cad

siE-1	-	-	+	+
Pyruvate	-	+	-	+

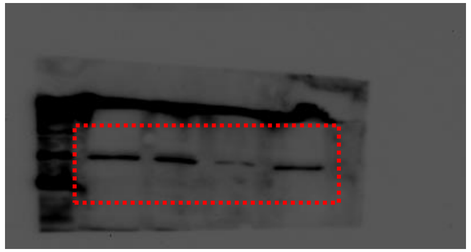

MMP9

siE-1	-	-	+	+
Pyruvate	-	+	-	+

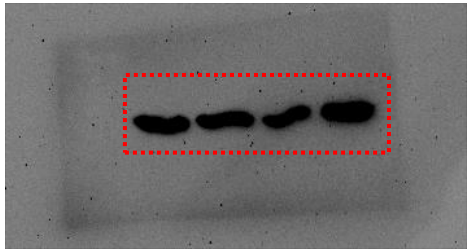

β-actin

siE-1	-	-	+	+
Pyruvate	-	+	-	+

Unedited gel in Figure 4E

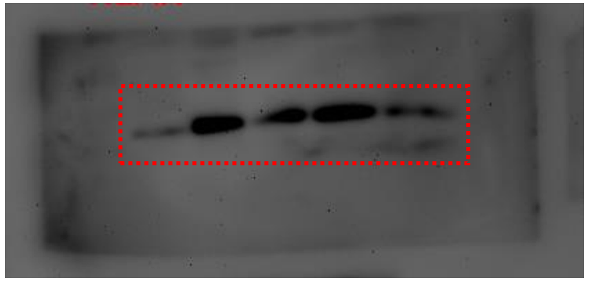

γH2AX

GEM - + + + +
Pyruvate (mM) - - 1 2 3

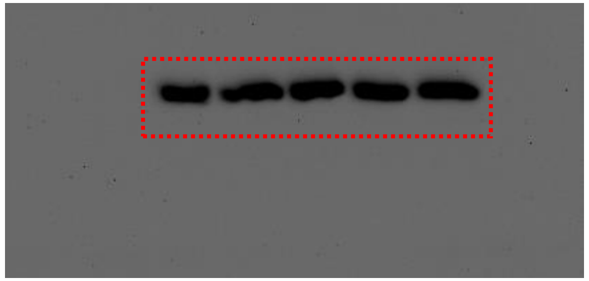

β-actin

GEM - + + + +
Pyruvate (mM) - - 1 2 3

Unedited gel in Figure 4G

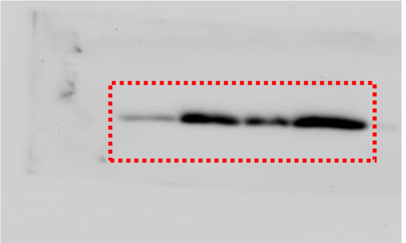

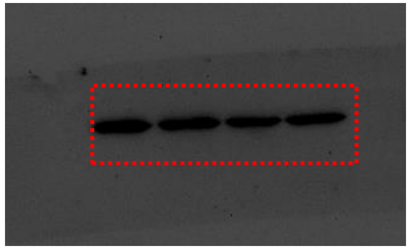

Unedited gel in Figure 4I

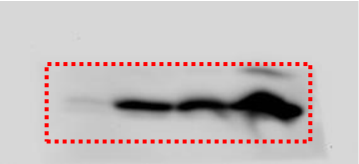

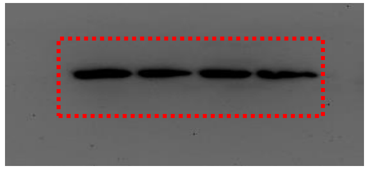

Unedited gel in Figure 6A

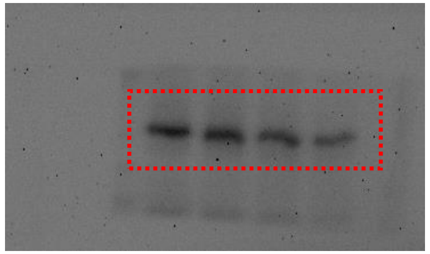

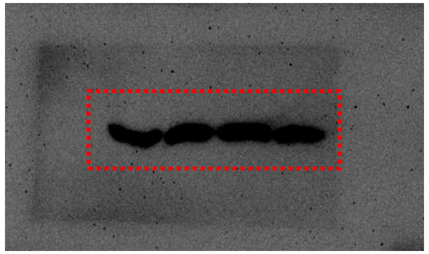

Unedited gel in Figure 6F

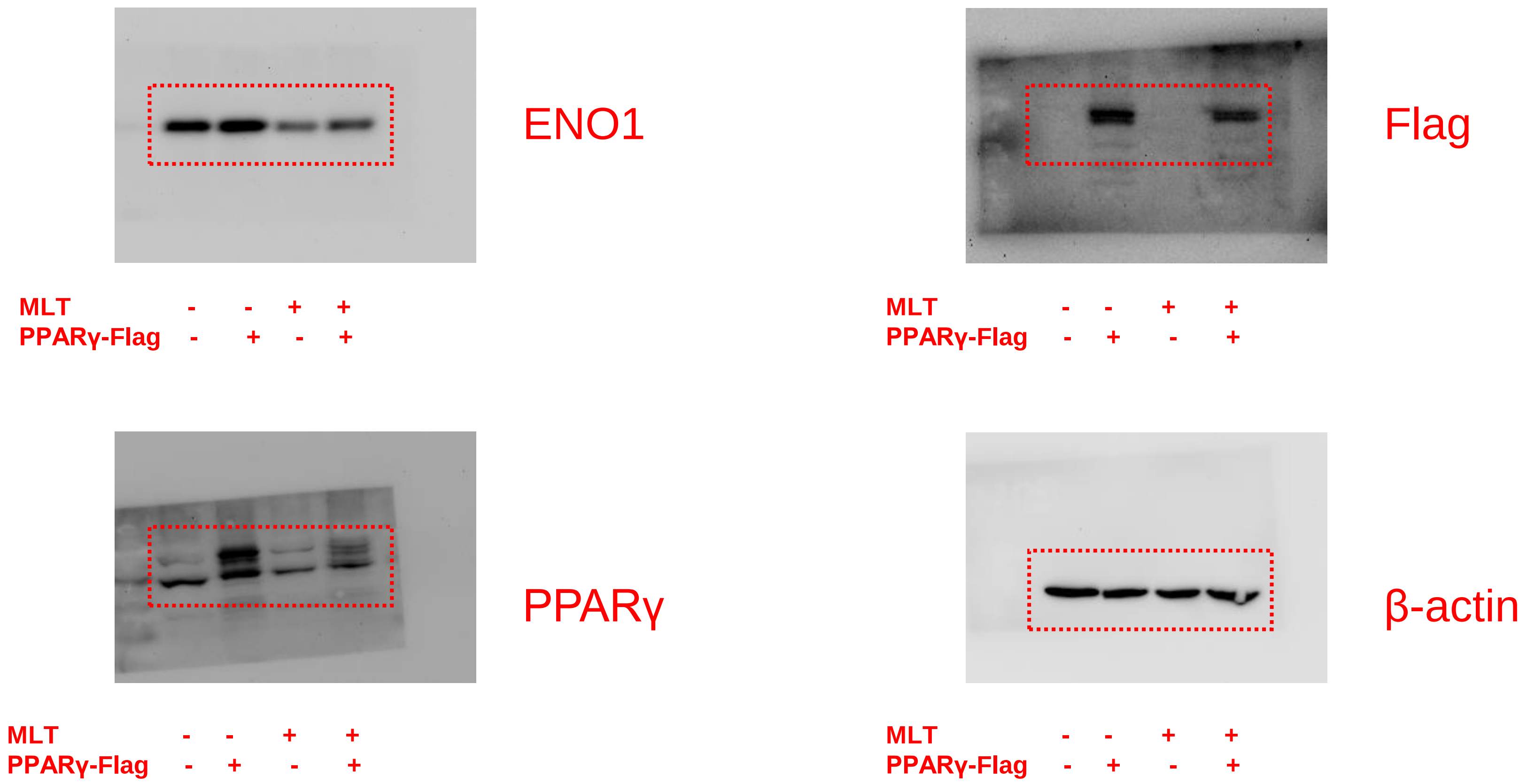

Unedited gel in Supplementary Figure S2E

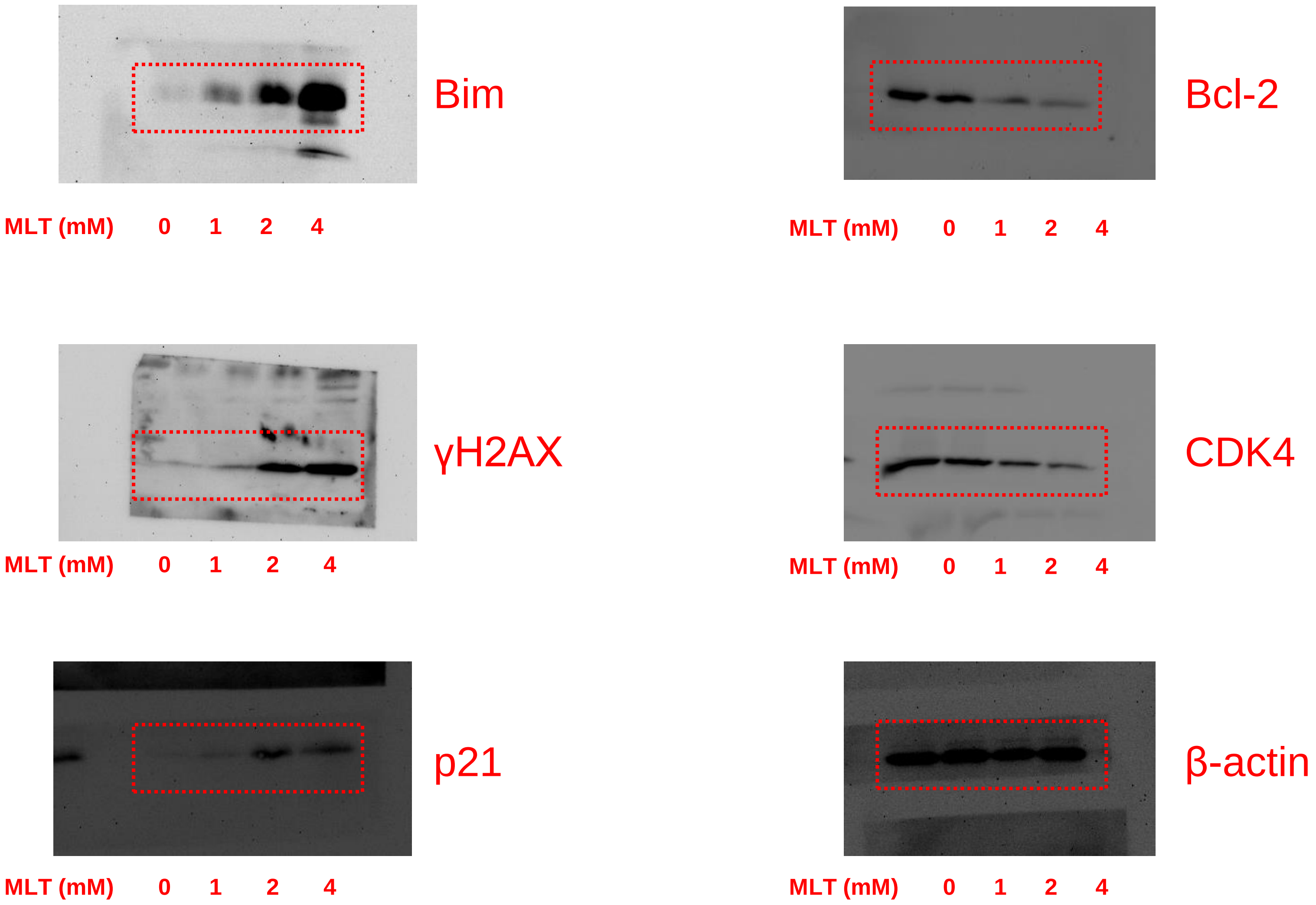

Unedited gel in Supplementary Figure S2F

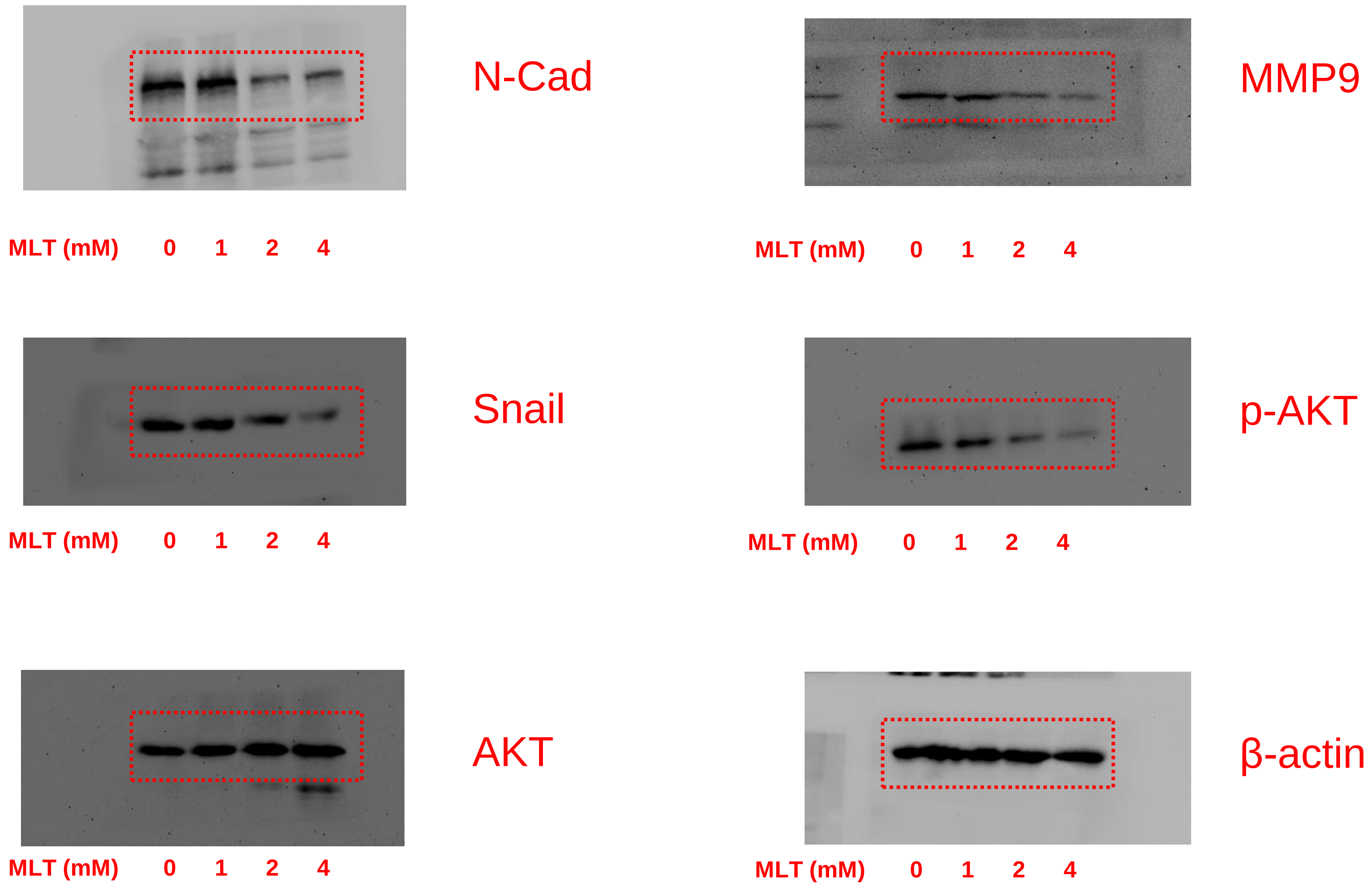

Unedited gel in Supplementary Figure S3B

5637

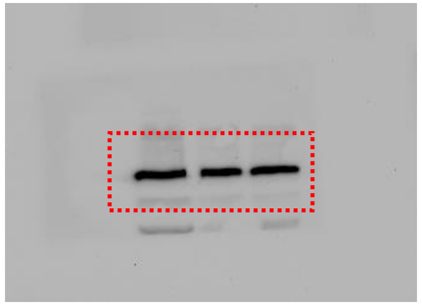

β-actin

NC siE-1 siE-2

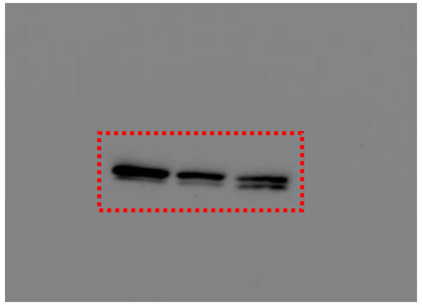

ENO1

NC siE-1 siE-2

T24

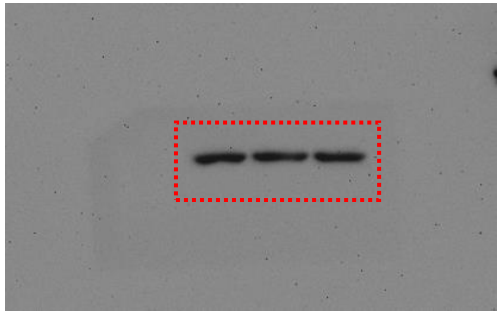

β-actin

NC siE-1 siE-2

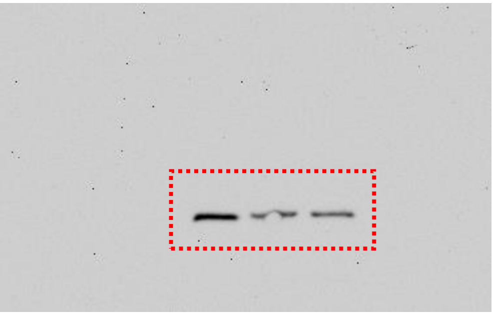

ENO1

NC siE-1 siE-2

UM-UC3

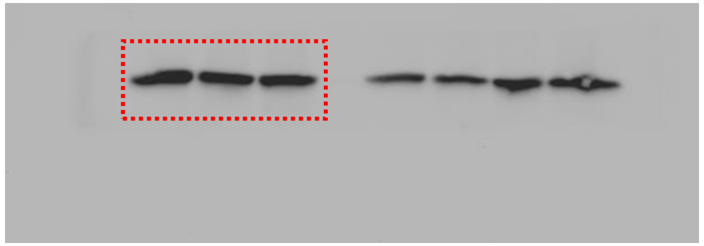

β-actin

NC siE-1 siE-2

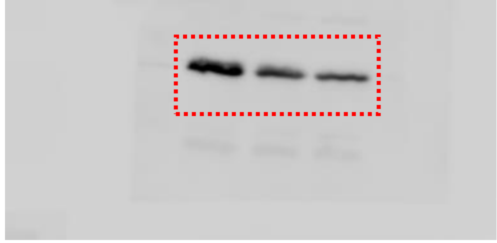

ENO1

NC siE-1 siE-2

Unedited gel in Supplementary Figure S4E

UM-UC3

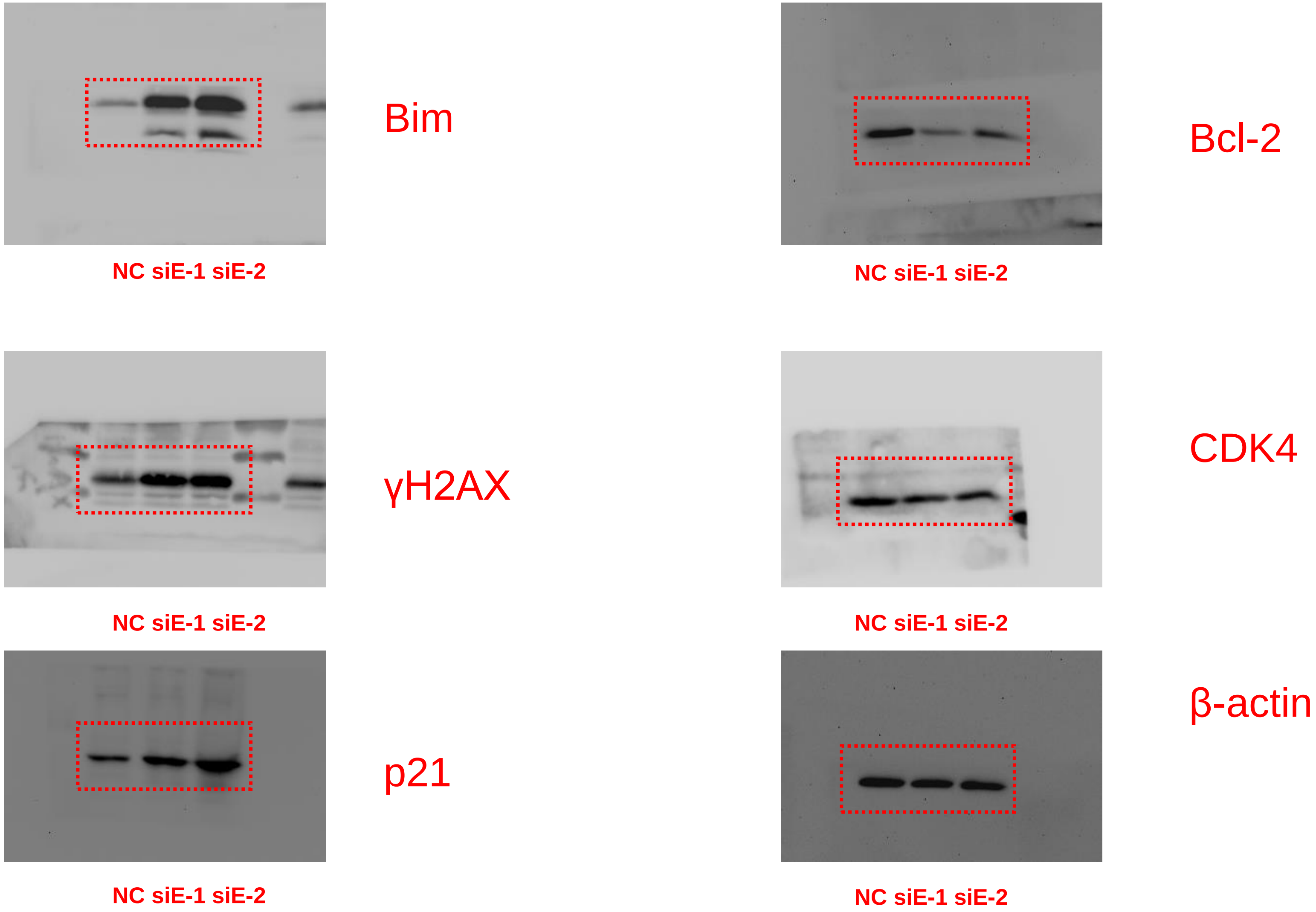

Unedited gel in Supplementary Figure S4E

T24

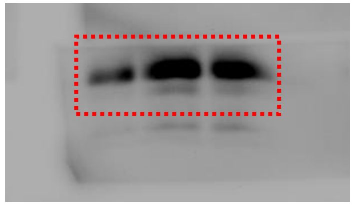

Bim

NC siE-1 siE-2

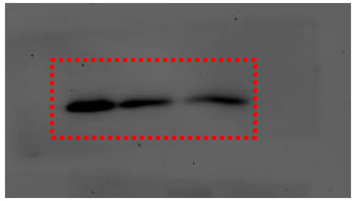

Bcl-2

NC siE-1 siE-2

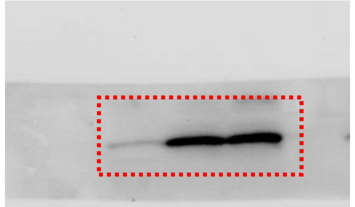

γH2AX

NC siE-1 siE-2

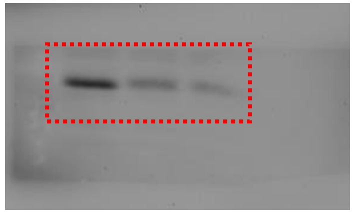

CDK4

NC siE-1 siE-2

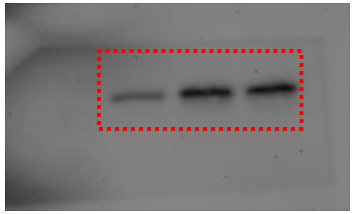

p21

NC siE-1 siE-2

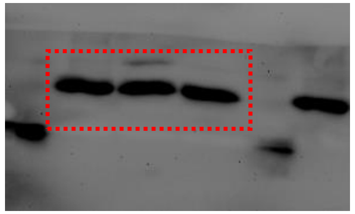

β-actin

NC siE-1 siE-2

Unedited gel in Supplementary Figure S4G

UM-UC3

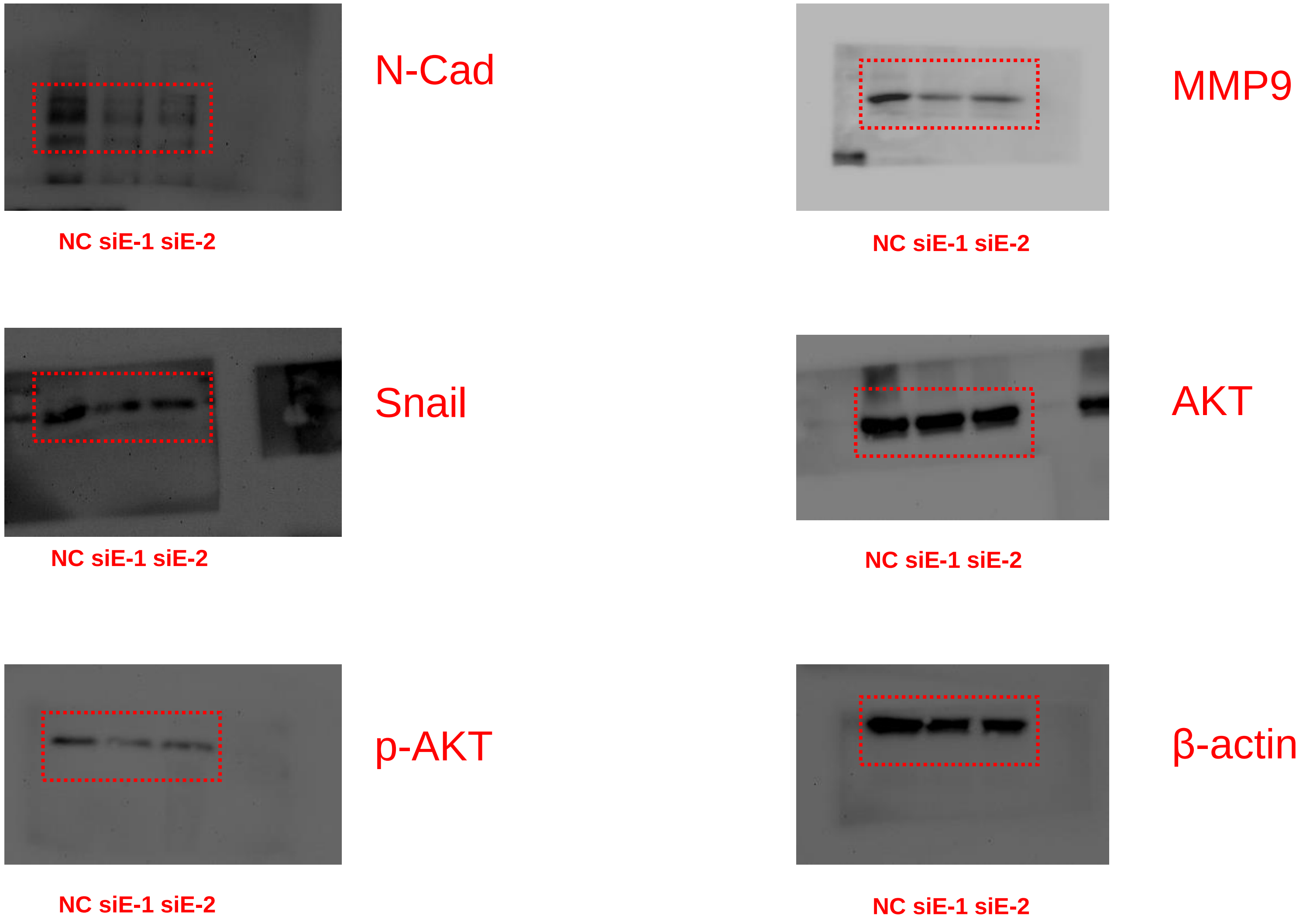

Unedited gel in Supplementary Figure S4G

T24

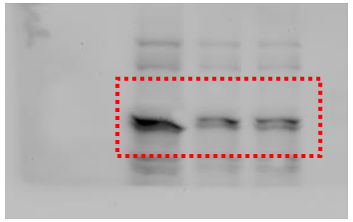

N-Cad

NC siE-1 siE-2

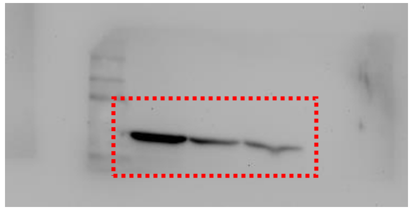

MMP9

NC siE-1 siE-2

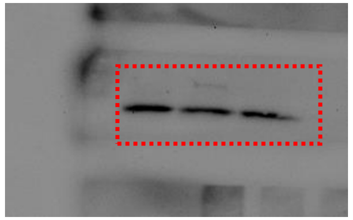

Snail

NC siE-1 siE-2

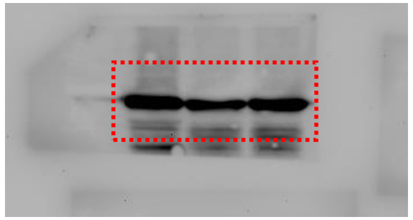

AKT

NC siE-1 siE-2

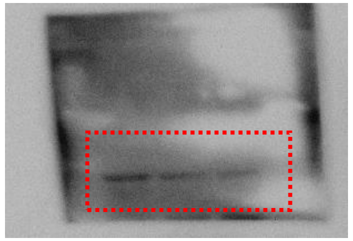

p-AKT

NC siE-1 siE-2

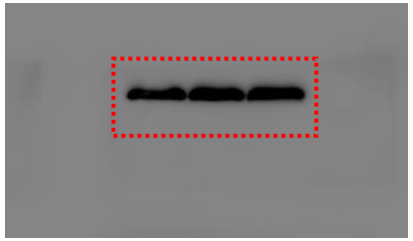

β-actin

NC siE-1 siE-2

Unedited gel in Supplementary Figure S5B

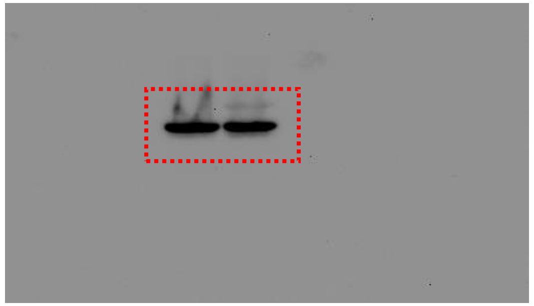

β-actin

Vec OE-ENO1

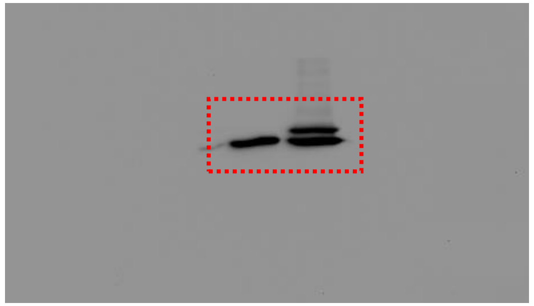

ENO1

Vec OE-ENO1

Unedited gel in Supplementary Figure S10B

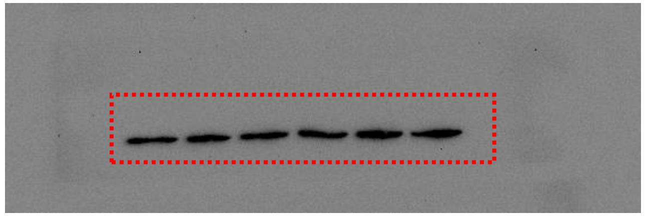

β-actin

Time (h) 0 2 4 8 12 24

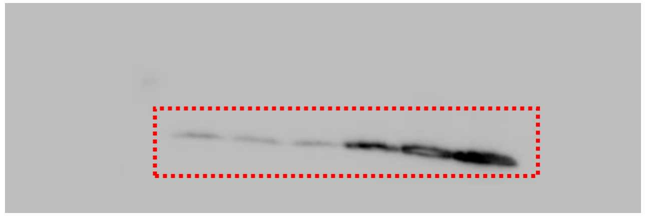

γ-H2AX

Time (h) 0 2 4 8 12 24

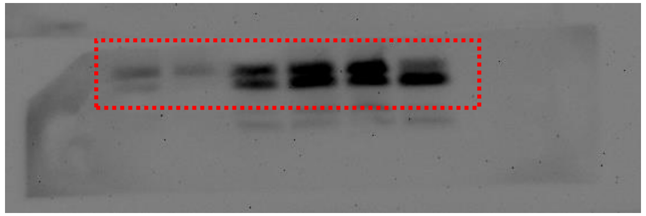

Bim

Time (h) 0 2 4 8 12 24

Unedited gel in Supplementary Figure S12B

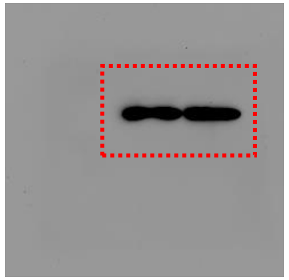

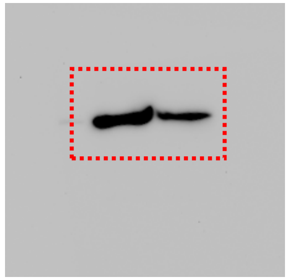

Unedited gel in Supplementary Figure S14C

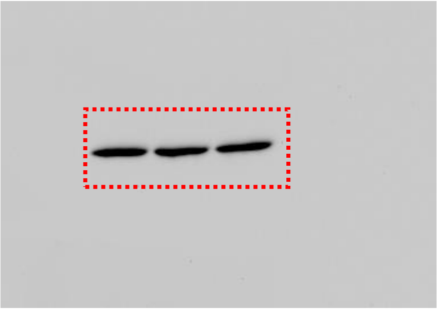

β-actin

NC siH-1 siH-2

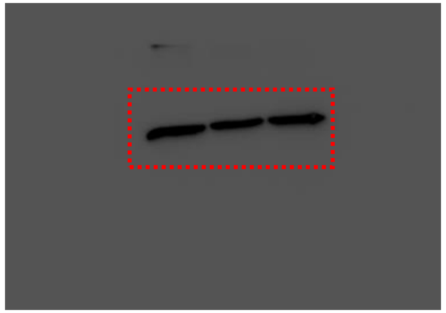

ENO1

NC siH-1 siH-2

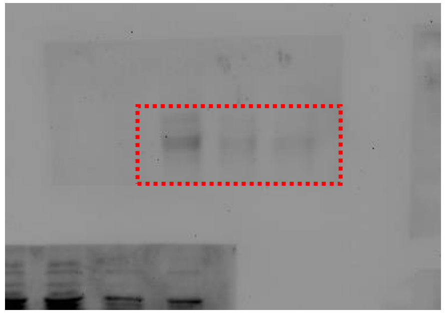

HIF-1α

NC siH-1 siH-2

Unedited gel in Supplementary Figure S14F

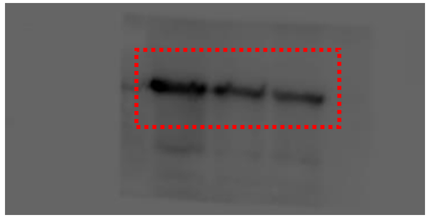

ENO1

NC siP-1 siP-2

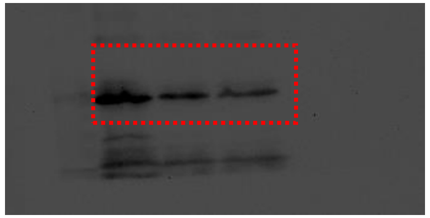

PPARγ

NC siP-1 siP-2

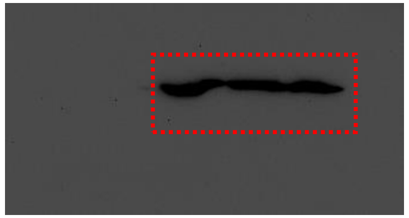

β-actin

NC siP-1 siP-2
